# Air Pollution, Asthma and Diet: From Mechanisms to Prevention Strategies

**DOI:** 10.3390/nu18040639

**Published:** 2026-02-15

**Authors:** Pedro Afonso Carvalho, Inês Paciência, André Moreira, Francisca de Castro Mendes

**Affiliations:** 1 Faculty of Medicine, University of Porto, 4200-319 Porto, Portugal; pedroattrc2002@gmail.com; 2 Center for Environmental and Respiratory Health Research (CERH), Research Unit of Population Health, University of Oulu, 90014 Oulu, Finland; ines.paciencia@oulu.fi; 3 Basic and Clinical Immunology Unit, Department of Pathology, Faculty of Medicine, University of Porto, 4200-319 Porto, Portugal; andremoreira@med.up.pt; 4 EPIUnit ITR, Instituto de Saúde Pública da Universidade do Porto, 4050-600 Porto, Portugal; 5 Faculty of Nutrition and Food Sciences, University of Porto, 4150-180 Porto, Portugal; 6 Serviço de Imunoalergologia, Centro Hospitalar São João, 4200-319 Porto, Portugal

**Keywords:** air pollution, asthma, diet quality, nutritional supplementation, effect modification

## Abstract

Background/Objectives: Air pollution is a major environmental determinant of asthma morbidity and lung function impairment across the life course. Both outdoor and indoor exposures contribute to asthma development and exacerbations, impaired lung function growth, and accelerated decline, with heightened susceptibility during pregnancy and childhood. In this narrative review, we aimed to: (i) synthesize evidence on outdoor and indoor air pollution in asthma and lung function decline; (ii) describe key modulators of pollution-related risk; (iii) evaluate diet and supplementation as effect modifiers; and (iv) outline strategies and recommendations to mitigate pollution-related asthma burden. Methods: A narrative synthesis was conducted based on a comprehensive PubMed literature search through 2025, integrating evidence from observational and interventional studies evaluating habitual diet and nutritional supplementation as potential modifiers of the respiratory effects of indoor and outdoor air pollution. Results: We synthesized human observational and interventional studies associating outdoor and indoor air pollution with asthma and lung function outcomes, highlighted major susceptibility modulators and mechanistic pathways, and appraised emerging evidence that habitual diet and nutritional supplementation might modify pollutant-related respiratory effects. Mechanistic evidence supported dietary modulation through redox buffering, epithelial-immune pathways, lipid-mediated inflammatory balance, and microbiome-immune crosstalk. However, human evidence remained heterogeneous across pollutants, settings, dietary metrics, and endpoints. Conclusions: Emissions reduction at source remained the cornerstone of prevention. Effective mitigation should be multi-level and equity-focused, combining structural air-quality improvements with pollution-aware asthma care and feasible household practices. Diet should be framed as a supportive, food-first resilience strategy, improving overall diet quality, fat quality, and fiber intake rather than a substitute for emissions reduction or guideline-based asthma management.

## 1. Introduction

Air pollution is a major global environmental determinant of respiratory health and remains a leading contributor to asthma morbidity and impaired lung function across the life course [1,2]. Short- and long-term exposure to outdoor pollutants, particulate matter (PM_2.5_ and PM_10_), nitrogen dioxide (NO_2_), and ozone (O_3_), has been consistently associated with asthma incidence and exacerbations, as well as impaired lung function growth and accelerated decline [3,4,5].

In addition to outdoor exposures, indoor air pollution represents a critical and often underappreciated contributor to asthma burden, especially in children, given the substantial proportion of time spent indoors at home and in school settings [6,7]. Indoor environments may contain complex mixtures of pollutants, including combustion-related particles, volatile and semi-volatile organic compounds (VOC and SVOC), endocrine-disrupting chemicals (EDC), dampness-related bioaerosols, and tobacco smoke, which have been associated with asthma symptoms, airway inflammation, and reduced lung function [6,7,8,9].

Against this background, diet has emerged as a biologically plausible and potentially modifiable factor that may influence susceptibility to the respiratory effects of air pollution, through its role in modulating systemic and airway inflammation, oxidative stress, and immune responses [10,11]. Observational studies suggest that healthier dietary patterns, including the Mediterranean diet, largely seen as a sustainable dietary pattern due to higher vegetable and reduced meat consumption, are associated with better asthma outcomes and lung function [12,13,14]. Conversely, Western-style dietary patterns characterized by higher intake of ultra-processed foods and therefore, less sustainable diets with higher environmental footprints and higher exposure to environmental pollutants accumulated within the food chain [15], associated with worse asthma outcomes and lung function [16]. From a One Health perspective, sustainable diets represent a co-benefit strategy, reduce air pollution by lowering food-system emissions and mitigate inflammation through the human–animal–environment interface [15]. Accordingly, growing evidence indicates that diet may act as an effect modifier of air pollution and asthma associations [17]. Moreover, experimental and clinical studies further support prior findings, demonstrating that specific dietary components and supplements, including antioxidants and omega-3 polyunsaturated fatty acids (ω-3 PUFA), can attenuate pollutant-induced airway inflammation and lung-function impairment [18,19]. However, evidence remains fragmented across pollutants, exposure settings, and dietary metrics, and translation into actionable prevention and policy recommendations is still limited.

Evidence for diet-by-pollution effect modification comes from two complementary lines: observational studies assessing interactions between habitual diet or nutrient status and outdoor/indoor pollution in relation to asthma incidence, symptoms, or lung function; and controlled exposure/supplementation studies, often in healthy volunteers or mild asthma testing whether specific nutrients attenuate acute pollutant-induced physiological or inflammatory changes. These designs address different endpoints and have different generalizability, which should be considered when interpreting findings and translating mechanistic evidence into clinical and public health recommendations.

The detrimental impact of air pollution on the respiratory system is well-established, as it remains a major global environmental determinant of asthma morbidity and lung function impairment across the life course. Despite the recognition of diet as a potentially modifiable factor influencing susceptibility to the respiratory effects of air pollution, existing evidence remains fragmented and across different pollutants, exposure settings, and dietary approaches and metrics. High-quality causal studies that explicitly investigate diet-by-pollutant interactions remain scarce and difficult to generalize. Furthermore, there is a notable gap in the translation of scientific findings into actionable clinical recommendations and public health policies.

In this narrative review, we therefore aimed to: (i) synthesize evidence on outdoor and indoor air pollution in asthma and lung function decline; (ii) describe key modulators of pollution-related risk; (iii) evaluate diet and supplementation as effect modifiers; and (iv) outline strategies and recommendations to mitigate pollution-related asthma burden.

## 2. Methods

This narrative review is based on a targeted literature search conducted in PubMed to identify relevant studies published up to 31 December 2025. Search terms combined key concepts related to both outdoor and indoor air pollution, asthma, lung function, diet and nutritional supplementation (e.g., “air pollution”, “particulate matter”, “nitrogen dioxide”, “ozone”, “indoor air”, “asthma”, “wheeze”, “lung function”, “diet”, “Mediterranean diet”, “supplementation”, “antioxidants”, “omega-3”). We prioritized human observational and interventional studies that evaluated habitual diet or nutritional supplementation as potential modifiers of respiratory outcomes related to indoor and/or outdoor air pollution, including airway inflammation, wheeze, lung function and asthma outcomes. Evidence was narratively synthesized and organized by exposure domain and by outcomes across the life course.

## 3. Outdoor and Indoor Air Pollution in Asthma and Lung Function

### 3.1. Life-Course Susceptibility and Developmental Windows

A consistent modulator of pollution-related asthma risk is the timing of exposure, with early-life windows showing stronger and more persistent associations than many later-life windows, plausibly due to rapid lung growth and immune maturation. Prenatal exposure to NO_2_, sulfur dioxide (SO_2_), and PM_2.5_ during fetal lung development has been associated with increased risk of childhood wheeze and asthma, highlighting the importance of early susceptibility windows [20,21,22]. Meta-analytical evidence indicates that each 5 μg/m^3^ increase in prenatal PM_2.5_ exposure is associated with a 6 to 10% higher risk of childhood asthma or recurrent wheeze [23]. Moreover, black carbon particles from urban air pollution have been detected on the fetal side of placentas, underscoring that inhaled pollutants can reach the fetus during pregnancy [24].

Evidence from longitudinal and multicohort studies further supports early-life sensitivity. In a large multi-cohort US analysis, early-life pollution averaged over the first three years of life was associated with incident asthma by early childhood (<5 years) and by middle childhood (<11 years) [25]. A 1-IQR increase in NO_2_ (6.1 μg/m^3^) was associated with higher asthma incidence both by <5 years (HR 1.25, 95% CI 1.03; 1.52) and by <11 years (HR 1.22, 95% CI 1.04; 1.44). Similarly, a 1-IQR increase in PM_2.5_ (3.4 μg/m^3^) was associated with higher asthma incidence by <5 years (HR 1.31, 95% CI 1.04; 1.66) and by <11 years (HR 1.23, 95% CI 1.01; 1.50).

Findings for prenatal O_3_ exposure are inconsistent. In one birth cohort, no association was observed between O_3_ and lung function in primary models [26]. Nevertheless, in a cross-sectional cohort study, each 10 μg/m^3^ increase in maximum daily 8 h average of daily O_3_ was positively associated with the risk of impaired lung function in childhood [27]. Accordingly, in a birth cohort, prenatal O_3_ exposure was associated with lower FEV_1_/FVC in childhood [28]. In another birth cohort, increased prenatal O_3_ exposure was positively associated with the risk of childhood wheezing throughout pregnancy and the second trimester with HRs of 1.22 (95% CI: 1.04–1.44) and 1.31 (95% CI: 1.09–1.58), respectively [29].

Evidence for prenatal CO exposure is more limited and sometimes attenuates after adjustment. In a nested case–control study, not as evidently as NO_2_, SO_2_ and PM_2.5_, as well as CO, were associated with increased risk in childhood asthma, but only on univariate analysis for 90 days before pregnancy and second trimester exposure, and not on multivariate analysis [30]. For whole-pregnancy exposure, a ~10% increase in risk was reported for transient tachypnea of the newborn [31], a condition associated with preschool asthma [32]. In a US cohort, prenatal CO exposure was negatively associated with FEF_25–75_ and early-life CO exposure had a negative effect on FEV_1_/FVC and (FEF_25–75_)/FVC, limited to subgroups of children with asthma [33].

A prospective birth cohort study including PM_2.5_ CO, SO_2_, NO_2_, and O_3_ reported that each quartile increase in second-trimester exposure to a mixed-pollutant index was associated with an adjusted HR of 1.24 (95% CI 1.04–1.47) for developing asthma/wheezing. Notably, CO contributed the most (64.28%) to the mutual effect [34]. Moreover, for the first year and first two years after birth, each quartile increment of exposure to mixed air pollutants was associated with HRs of 1.65 (95% CI 1.30–2.10) and 2.53 (95% CI 2.16–2.97), respectively for developing asthma or wheezing. Notably, SO_2_ contributed the most in both phases, accounting for 50.30% and 74.70% of the association, respectively [34].

### 3.2. Life-Course Timing and Developmental Windows

Epidemiological studies have shown that long-term exposure to traffic-related air pollution (TRAP), including PM and NO_2_, is associated with new-onset asthma in children [25]. Global analyses estimate that approximately 13% of pediatric asthma incidence, over 1.8 million cases in 2019, may be attributable to ambient NO_2_, with the highest burdens in densely NO_2_-polluted cities [35]. Consistently, in multi-cohort analyses, incremental increases in early-life PM_2.5_ and NO_2_ exposure have been associated with an approximately 20–30% higher asthma incidence during childhood [25].

Short-term increases in ambient O_3_ can precipitate asthma exacerbations, as observed in time-series and panel studies in both pediatric and adult populations with asthma [36]. In addition to acute morbidity, chronic O_3_ exposure has been linked to lasting impairment in lung function among adults. In the European Community Respiratory Health Survey (ECRHS), long-term O_3_ exposure was associated with faster declines in FEV_1_ and FVC over approximately 20 years, with estimates robust to adjustment for smoking and other relevant confounders and co-pollutants [37].

However, evidence for long-term O_3_ effects on lung function in childhood is more mixed; in a cohort with relatively low O_3_ concentrations, O_3_ exposures averaged from birth to age eight were not associated with adverse lung function in middle childhood [38].

A narrative review including systematic reviews and meta-analysis stated that no association was found between CO and O_3_ and the asthma development in children, contrasting with NO_2_, PM and SO_2_ [39].

Nevertheless, several studies have linked CO exposure to poorer lung function. In a four-year clinical trial of children with asthma aged five to 12 years, short-term exposure to CO was negatively associated with post-bronchodilator %predicted FEV_1_, and long-term exposure (up to 4 months) was negatively associated with prebronchodilator %predicted FEV_1_ and FVC [40]. CO and O_3_ four-month exposures were negatively associated with FEV_1_/FVC [40]. In a panel study of adults over two years, an increase in CO was associated with decreased lung function, especially a decrease in PEF [41].

A systematic review and meta-analysis of observational studies found that incremental increases in outdoor air pollutants such as NO_2_, O_3_, CO, and TRAP contribute to adolescent asthma [42]. For CO, includes four cross-sectional studies and one cohort (two-year panel study), being that the cohort does not find association between CO exposure and current asthma [42].

CO_2_ is commonly used as an indicator of ventilation adequacy and occupant density in indoor environments [43]. Accordingly, associations between indoor CO_2_ and asthma-related outcomes are inconsistent and may reflect co-exposures (e.g., indoor pollutants accumulating under poor ventilation) rather than a direct toxic effect at typical concentrations. Specifically, a cross-sectional study in nurseries and primary schools indicated that CO_2_ was not significantly associated with the increase in the odds of having any of the respiratory asthma outcomes in study [44]. However, CO_2_ > 2100 ppm values were associated with exercise-induced wheeze [OR = 1.86 (95% CI: 1.20–2.89)] and night cough [OR = 1.40 (4.20–2.89)] [45] and in a case–control study in 454 children, only in winter, indoor CO_2_ concentration were significantly associated with the increased odds of childhood asthma [46].

### 3.3. Policy-Driven Air Quality Improvements and Respiratory Improvements

By contrast, improvements in ambient air quality have demonstrable benefits for respiratory health [47]. One of the clearest demonstrations comes from the Southern California Children’s Health Study, which compared three cohorts of children followed from age 11 to 15 years during a period of sustained regional air-quality improvement [47]. Across communities, declines in key pollutants were associated with larger four-year gains in lung function. Specifically, a 14.1 ppb decrease in NO_2_ was associated with an additional 91.4 mL in FEV_1_ growth over four years (*p* < 0.001), while an 8.7 μg/m^3^ decrease in PM_10_ and a 12.6 μg/m^3^ decrease in PM_2.5_ were each associated with approximately 65.5 mL greater FEV_1_ growth (PM_10_: *p* < 0.001; PM_2.5_: *p* = 0.008). Changes in O_3_ exposure were not significantly associated with lung-function growth. These improvements were mirrored in FVC growth, reinforcing that cleaner air supports broader lung development during a critical window of respiratory maturation [47,48].

Decreases in the levels of NO_2_, PM_2.5_, PM_10_, and O_3_ were associated with decreased prevalence of respiratory symptoms (bronchitis, cough, and phlegm), particularly in children with asthma [48].

Importantly, these findings were clinically relevant at the population level: the proportion of adolescents with clinically low lung function by age 15 declined across successive cohorts exposed to improved air quality [47]. The Health Effects Institute’s policy-focused synthesis further notes that these gains were observed in both boys and girls and among children with and without asthma, supporting the relevance of pollution reduction both for children living with asthma and for primary prevention [48].

### 3.4. Indoor Air Pollution: Sources, Chemical Mixtures, and Asthma Morbidity

Indoor air pollution plays a major role in asthma development and morbidity. Common indoor pollutants include environmental tobacco smoke, emissions from biomass fuel combustion, nitrogen oxides from gas stoves, PM generated by cooking and indoor activities, as well as VOC and SVOC released from building materials, furnishings, and cleaning products [6,7]. Exposure to these indoor contaminants has been associated with both the development of asthma and the worsening of existing disease [9].

Some indoor VOC and SVOC can also act as EDC, and have been associated with respiratory symptoms and asthma in epidemiological studies [6,49]. In this context, a cross-sectional study in school-aged children found that even at relatively low concentrations, increased classroom levels of certain VOCs (e.g., aromatic hydrocarbons) increased the odds of having asthma and obesity, suggesting that these exposures may affect metabolic and respiratory health concurrently [6]. Likewise, exposure to indoor formaldehyde and other irritant gases can provoke airway inflammation and have been correlated with asthma-like symptoms in children, with meta-analytical evidence supporting associations with asthma outcomes [50].

Moreover, indoor allergens and microbes can interact with pollution. Specifically, TRAP can attach to pollen or dust, potentially enhancing allergenicity, and NO_2_ can increase airway responsiveness to inhaled allergens [51].

Cord blood samples from several birth cohort studies suggest that prenatal NO_2_ exposure is associated with DNA methylation in several mitochondria-related genes and involved in antioxidant defense pathways, contributing to oxidative stress [52]. Evidence also supports time-sensitive developmental Windows. Specifically, in a Swedish birth cohort NO_2_ exposure in the first year of life was associated with increased risk of pollen sensitization at age four, whereas associations were less clear at age eight, highlighting heterogeneity by age and outcome definition [53]. This aligns with life-course timing and developmental windows, as long-term exposure to TRAP is associated with new-onset asthma in children [25].

In addition to longer-term programming effects, common pollutants can acutely exacerbate respiratory symptoms. NO_2_ and PM, along with SO_2_ and O_3_, may act as airway irritants that can induce cough, increased mucus hypersecretion, and bronchial hyperresponsiveness, contributing to a greater number of emergency department visits for asthma and other respiratory complaints [51].

Importantly, co-exposures may operate through bioaerosol interactions. PM, PAHs, NO_2_, SO_2_, and CO may modify the surface and biological properties of atmospheric bioaerosols, such as pollen and fungi, thereby enhancing their allergenic potential [54]. This co-exposure may facilitate deeper penetration of fungal allergens and skew the immune response toward pro-inflammatory Th2 and Th17 phenotypes, creating a ‘double hit’ that amplifies airway inflammation [51]. For example, in a cohort study in California, exposure to increased daily levels of basidiospores and ascospores in the first three months of life was associated with increased odds of wheezing among children under two years [55]. Indoor mold exposure may initiate asthma and influence asthma outcomes through both IgE- and non-IgE-mediated mechanisms, especially among children [56]. Accordingly, mold removal could reduce the negative impact of indoor air pollution on asthma through improving asthma symptoms and reduced medication [57]. According to EAACI Guidelines, mold exposure had a moderate certainty of evidence for new-onset asthma [9].

### 3.5. Biological Pathways Linking Pollution Exposure to Asthma Heterogeneity

From a biological perspective, air pollution contributes to asthma pathophysiology through a complex interplay of epithelial barrier disruption, oxidative stress, and immune dysregulation, activating multiple inflammatory endotypes beyond classical type 2 responses [9,58]. Pollutant exposure can induce epithelial injury and innate immune activation, promoting type 3 (Th17/IL-17-driven) and mixed inflammatory patterns characterized by neutrophilic airway inflammation, as well as metabolic–immune dysregulation involving inflammasome activation and systemic low-grade inflammation [59]. These pathways intersect with oxidative stress responses and altered lipid and glucose metabolism, which may further amplify airway hyperresponsiveness and lung-function impairment, particularly in susceptible phenotypes [60,61]. Clinically, these mechanisms are relevant because non-type 2 and neutrophilic asthma phenotypes, as well as metabolically driven inflammatory patterns, tend to respond poorly to inhaled corticosteroids, highlighting an unmet need for complementary strategies capable of targeting oxidative stress, innate immune activation, and metabolic inflammation in pollution-related asthma [60,61,62].

While air pollutants share common pathways, such as oxidative stress, they exhibit distinct biological signatures driven by their physical properties and deposition patterns. O_3_ and NO_2_ are low-solubility gases that penetrate deeply into the distal lung [51]. However, O_3_ specifically targets epithelial barrier integrity by altering claudin expression, whereas NO_2_ acts as a potent immunological adjuvant, upregulating ICAM-1 and facilitating allergen sensitization [56]. In contrast, SO_2_ is highly water-soluble and primarily triggers sensory receptors in the upper airways, inducing rapid, reflex-mediated bronchoconstriction [51,56]. PM functions as a polytoxic vector, where its impact is size-dependent: PM_10_ deposits in the primary bronchi, PM_2.5_ reaches the alveoli, and ultrafine particles (PM_0.1_) can penetrate the air-blood barrier to cause systemic toxicity [51,56]. Finally, CO induces systemic hypoxic stress via high-affinity hemoglobin binding, leading to tissue hypoxia rather than direct mucosal irritation [51,56].

### 3.6. Climate Change, Extreme Events and Emerging Pollutants on Asthma

Climate change, enhanced by anthropogenic activity is one of the main contributors to health emergencies worldwide being closely interrelated with CO_2_, NO_x_ and black carbon (naturally occurring greenhouse gases), and especially affecting children [63]. 

Climate change acts as a fundamental “threat multiplier,” shifting the epidemiology and severity of asthma, wheezing, and lung function impairment through both direct and indirect pathways [63,64].

Extreme temperatures, including heatwaves and cold spells, are robustly associated with an increased risk of asthma-related emergency department visits and hospital admissions with low certainty evidence [65], even though this was especially verified if exposed to heat for several days [66]; and in males and depending on age, those less than or four years old and those aged 10–14 were more susceptible to heat and cold, respectively [67]. Meta-analytic estimates suggest that heatwaves increase the risk of asthma ED visits by 34% (RR 1.34), while cold spells can nearly double this risk (RR 1.84) [65]. Furthermore, temperature variability (day-to-day variance) has also been recognized as a significant risk factor for acute respiratory infections and wheezing in children, particularly those under five years of age [63].

Global warming accelerates plant growth and prolongs pollen seasons, increasing human exposure, triggering early-life symptomatic sensitization, and putting asthmatic children at risk of exacerbation [63,64]. Specific phenomena, such as “thunderstorm asthma,” result in severe bronchospasm when pollen grains rupture due to osmotic shock, releasing allergenic particles deep into the lower airways [63,64]. Additionally, extreme events such as wildfires can release high concentrations of PM_2.5_, which are strongly associated with persistent asthma symptoms and hospitalizations [64]. Similarly, intense rainfall and flooding facilitate the proliferation of indoor molds (e.g., *Alternaria* and *Cladosporium*), which is associated with a 1.4- to 2.2-fold higher risk of asthma and wheezing in children [64].

The modern exposome also includes emerging chemical and biological threats.

For example, outdoor pesticide exposures, including fumigants and organophosphates have been associated with increased asthma incidence and worse asthma control [65]. Indoors, new VOCs play an important role in airway inflammation [43], as discussed in Section 3.4. Collectively, these anthropogenic shifts contribute to a substantial loss of environmental and human microbiome biodiversity, a reduction linked to impaired immune tolerance and a skewing toward pro-inflammatory Th2 and Th17 allergic responses [64]. These evolving challenges reinforce the need for strategies that integrate planetary health with individual respiratory resilience.

## 4. Modulators of Air Pollution and Asthma Risk

Air pollution does not affect all individuals equally. Inter-individual heterogeneity reflects differences in exposure (where and how people live) and differences in susceptibility (host biology, comorbidities, and co-exposures).

### 4.1. Socioeconomic Context and Environmental Inequality

Beyond timing, the burden of pollution-related asthma is heavily stratified by socioeconomic and neighborhood factors, a phenomenon commonly described as environmental inequality [68]. Environmental inequality refers to the systematic and disproportionate exposure of socioeconomically disadvantaged populations to environmental hazards (including air pollution), combined with greater vulnerability to their health effects due to structural and social determinants [69]. Consequently, low-income and marginalized communities often experience both higher pollutant levels and more severe asthma-related outcomes at comparable exposure intensities [70].

Evidence from Allegheny County using data from the AIR registry and a Pennsylvania Environmental Justice (EJ) tract definition (≥30% non-White residents and/or ≥20% living in poverty) found that children and adults residing in EJ tracts were substantially more likely to experience higher levels of TRAP than those in non-EJ areas [70]. Specifically, 66.4% of EJ-tract residents were classified in the highest NO_2_ exposure quartile, compared with 20.8% of non-EJ residents, and 41.6% were in the highest quartile of black carbon exposure versus 17.0% among non-EJ residents. Another clear example comes from a multicohort analysis in which early-life exposure to ambient pollution was associated with increased childhood asthma incidence, with effect estimates that differed across socially patterned strata [25]. For the same increase in PM_2.5_ exposure, the hazard ratio (HR) for asthma incidence by age five was 1.60 (95% CI 1.15; 2.22) among Black children, whereas the corresponding estimate among White children was 1.17 (95% CI 0.90; 1.52), indicating the heterogeneity of effects across population groups.

Neighborhood-level injustice indices also map onto asthma morbidity and lung function trajectories [71]. In metropolitan Atlanta, children from high-injustice census tracts had more asthma exacerbations, shorter time to first exacerbation, persistently more symptoms, poorer asthma control, and reduced lung function over follow-up compared with peers from lower-injustice tracts [71]. Moreover, living in the highest NO_2_ exposure quartile (Q4 vs. Q1–Q3) was associated with higher odds of uncontrolled asthma (OR 3.54, 95% CI 1.01; 12.39) [70].

A pediatric-focused review frames environmental inequality in childhood asthma as a combination of unequal pollutant exposures often driven by urban planning, transport patterns, and segregation, together with unequal capacity to avoid exposures and to manage disease, which may sustain asthma disparities even when overall air quality improves [6]. Mechanistically, this “inequality effect” is plausibly explained by a double or multiple-hit model: disadvantaged neighborhoods often experience higher traffic and/or industrial emissions and higher indoor co-exposures, alongside higher psychosocial stress [72,73], dietary constraints [74], comorbidities such as obesity [75], and barriers to consistent asthma care [76]. Together, these factors can amplify pollutant-induced oxidative and inflammatory injury and contribute to persistent disparities in asthma morbidity and lung function [6].

Evidence for CO, O_3_, and CO_2_, is discussed in other sections where relevant; in this section, we focus on pollutants and study designs directly aligned with the specific exposure–modifier–outcome framework.

### 4.2. Obesity and Metabolic Factors

Obesity and related metabolic disorders are increasingly recognized as important treatable traits that amplify asthma risk and severity [62]. Patients with obesity and asthma consistently show poorer disease control, greater severity, and impaired lung function compared with individuals with asthma and normal weight [77]. Beyond its direct clinical impact, growing evidence suggests that obesity may modify susceptibility to indoor and ambient pollutants, amplifying their adverse effects on asthma outcomes [78].

Both prenatal and postnatal exposure to secondhand smoke (SHS) have been robustly linked to childhood asthma, and several studies indicate that obesity may exacerbate SHS-related respiratory morbidity [79]. In two prospective studies including predominantly urban, African American school-aged children, obesity intensified the association between SHS exposure and asthma symptoms [80]. While SHS exposure was associated with worse asthma outcomes overall, symptom burden was greatest among children with obesity. These findings are supported by population-based evidence: SHS exposure at home was associated with a markedly higher prevalence of current asthma among adolescents with obesity (OR 2.01, 95% CI 1.15; 3.70), whereas no association was observed among non-obese peers (OR 0.94, 95% CI 0.71; 1.23) [81]. Similarly, regarding indoor exposure to polycyclic aromatic hydrocarbons (PAHs), a cohort of inner-city children from New York City and the Bronx showed that higher residential indoor levels of methyl-phenanthrenes increased the odds of current asthma at ages five and seven, but only among children with obesity [82].

School environments represent another important indoor microenvironment for children. In a one-year prospective study of children aged four to 13 years, classroom NO_2_ levels were associated with more asthma symptom days (IRR 1.86, 95% CI 1.15; 3.02) and greater disruption of caregiver daily plans among children with obesity, but not among children with normal weight [83]. Although evidence on interactions between obesity and indoor chemical mixtures, including EDC, remains limited, higher classroom levels of VOC have been associated with both obesity and asthma in school-aged children [6]. Specifically, higher classroom concentrations of toluene, o-xylene, m/p-xylene, ethylbenzene, and benzene were associated with 43% higher odds of asthma in the presence of obesity (OR 1.43, 95% CI 1.01; 1.98). In parallel, higher classroom concentrations of hexane, cyclohexanone, styrene, butylated hydroxytoluene, and 2-butoxyethanol were associated with overweight (OR 1.51, 95% CI 1.28; 1.79).

Obesity may also increase susceptibility to ambient pollutants by altering respiratory physiology and particle deposition. A recent study showed that children with obesity had significantly higher tidal volumes and minute ventilation, which may increase pulmonary deposition of inhaled fine particles [84]. On average, compared with normal-weight peers, children with obesity had a 25 mL higher tidal volume (95% CI 5; 45 mL) and a 453 mL/min higher minute ventilation (95% CI 123; 784), alongside a 3.4% greater alveolar deposition fraction of PM_2.5_ (95% CI 1.3; 5.5%) [84]. In practical terms, each breath of polluted air may deliver more particles deep into the lungs of children with obesity, providing a plausible explanation for the synergistic effects of obesity and pollution on asthma morbidity [84].

### 4.3. Natural Environment and Biodiversity

Features of the natural environment may modulate asthma risk and influence susceptibility to air pollution across the life course. The biodiversity hypothesis proposes that reduced contact with diverse environmental microbiota driven by urbanization, land-use change, and loss of green space may impair the development of immune tolerance, thereby increasing the risk of allergic diseases, including asthma [85]. In addition to immunologic pathways, green infrastructure may also influence local pollution exposure (e.g., proximity to traffic, dispersion and microclimate), although effects are context-dependent and can vary by vegetation type and setting.

Early insights into this association emerged from consistent observations that children raised in traditional farming environments experience substantially lower rates of asthma and allergy than their urban counterparts—an effect that cannot be explained by single exposures alone [86]. Rather, these environments provide sustained, complex microbial inputs that shape immune maturation during critical developmental windows. Children growing up in rural or farming settings exhibit enhanced activity of immune regulatory pathways, including increased expression of anti-inflammatory mediators and receptors involved in immune tolerance, suggesting long-term imprinting of the immune system [87,88,89].

Importantly, protection is not strictly tied to farming as a lifestyle. Evidence indicates that the microbial richness of the residential environment itself—particularly indoor house dust—predicts allergy-preventive effects even in non-farming homes, supporting the idea that microbial exposure, rather than rural residence per se, is a key determinant [90]. Within urban populations, characteristics of the surrounding environment, such as greenness and biodiversity, are reflected in skin microbiota composition and immune markers associated with allergy risk, reinforcing the relevance of everyday environmental contact [86]. Evidence from birth cohorts highlights the context-dependent nature of these effects. In the Portuguese Generation XXI cohort, residing in neighborhoods surrounded by more vegetation at age 10 was associated with a lower risk of allergic sensitization to food and aeroallergens [91]. In the same cohort, increasing close-proximity greenness around residences was also associated with higher levels of FEV_1_ and FEF_25–75_ [92]. Similarly, in a large national birth cohort from New Zealand, higher residential greenness and greater diversity of natural vegetation were independently associated with a lower risk of childhood asthma up to 18 years of age [93]. Consistent with the broader framework linking urban environmental change to allergic outcomes, findings from the COPSAC2010 prospective birth cohort showed that increasing urbanization was associated with a higher risk of childhood asthma, eczema, and allergic sensitization by age six [94]. Moreover, a longitudinal Finnish study found that favorable changes in neighborhood characteristics, including improvements in green space availability and socioeconomic indicators were associated with reduced all-cause mortality and lower incidence of multiple chronic diseases [95].

Evidence in adults also suggests potential benefits beyond primary prevention. More frequent use of green spaces has been associated with lower asthma medication use, pointing to a possible role of natural environments in modulating disease control [96], although causality remains difficult to establish [97]. At the same time, exposure to green environments may carry risks in certain contexts. High pollen loads, particularly during sensitive windows such as pregnancy or early childhood, have been linked to increased asthma risk later in life, underscoring the need to balance ecological benefits with allergenic potential when designing nature-based interventions [98].

## 5. Effect Modification of Diet and Nutritional Supplementation on Air Pollution

### 5.1. Mechanisms Underlying Dietary Effects

Dietary intake is a modifiable exposure that may modify susceptibility to air pollution (i.e., act as an effect modifier) by shaping baseline oxidative stress, inflammation, and immune regulation in asthma [61]. Air pollutants, including PM and O_3_, induce reactive oxygen species (ROS) generation, deplete intracellular antioxidant defenses, such as glutathione, and trigger downstream cytokine and chemokine production, nutritional exposures that influence antioxidant capacity or inflammatory signaling are biologically plausible effect modifiers [17]. Within this framework, a pollution-protective dietary profile is hypothesized to reduce pollutant-triggered airway injury by attenuating oxidative stress, dampening innate immune activation, and limiting exaggerated recruitment and activation of effector cells, including neutrophils [61]. However, mechanistic plausibility does not necessarily translate into proven clinical benefit, and the strength of evidence differs across endpoints and study designs.

A central mechanistic axis is redox buffering, whereby diets rich in antioxidant micronutrients and phytochemicals may neutralize oxidant burden and/or upregulate endogenous antioxidant pathways, thereby reducing pollution-induced epithelial injury and inflammatory amplification [18]. In pregnancy cohorts, higher composite antioxidant intake derived from vitamins, carotenoids, and trace minerals may attenuate prenatal PM_2.5_-associated wheeze risk in susceptible subgroups [99], aligning with an antioxidant-mediated modification hypothesis. At the dietary-pattern level, greater vegetable diversity can serve as a proxy for broader exposure to fiber, carotenoids, polyphenols, and other bioactive compounds that jointly support antioxidant and anti-inflammatory activity [14], potentially lowering baseline airway inflammation and reducing vulnerability to pollutant triggers. Notably, these effects are expected to be food matrix- and synergy-driven, arising from additive or complementary actions of multiple compounds rather than a single nutrient in isolation [14], which may help explain heterogeneity across supplementation trials.

Accordingly, high-dose supplementation under high pollution exposure may not uniformly benefit and may even show pro-oxidant patterns in some contexts, reinforcing that dose, baseline status, and exposure context are mechanistically important [61]. Cruciferous-derived isothiocyanates (e.g., sulforaphane) may also upregulate endogenous antioxidant defenses via NRF2-related pathways, providing additional mechanistic rationale for attenuation of pollution-induced oxidative injury [100]. Vitamin D-responsive epithelial-immune and redox pathways may provide complementary mechanistic plausibility, particularly under high oxidative burden conditions. Beyond its endocrine roles, vitamin D signaling can influence airway epithelial barrier integrity and immune regulation, and experimental work in human bronchial epithelial cells suggests that vitamin D metabolites may attenuate PM-induced inflammatory and oxidative stress responses, including increased expression of antioxidant pathway genes, such as G6PD, shifts in glutathione redox balance, and reduced oxidative damage markers [101,102]. More recent experimental data additionally indicate protective effects on PM-induced mitochondrial injury and calcium dyshomeostasis in bronchial epithelial models, supporting a broader cytoprotective rationale [101]. Together, these in vitro findings support the hypothesis that vitamin D sufficiency could protect against pollutant-triggered epithelial injury and inflammatory amplification, although such effects are expected to be context-dependent and do not, by themselves, establish clinical benefit [102].

A second key axis is diet–microbiome–immune crosstalk, whereby fermentable fiber increases short-chain fatty acid (SCFA) production that can modulate immune responses via G-protein-coupled receptor (GPCR) signaling (e.g., GPR41/GPR43) and epigenetic regulation, including histone deacetylase (HDAC) inhibition, with downstream effects on airway inflammation relevant to pollutant-induced neutrophilic biology [61]. Small human studies suggesting that increased fiber intake can rapidly reduce airway inflammation, alongside changes in GPR43/GPR41 expression, support a mechanistic pathway through which dietary fiber could plausibly buffer acute inflammatory responses to inhaled pollutants [103]. Complementary trial data indicate that short-term soluble fiber supplementation (±probiotic) can shift airway inflammatory markers in asthma [104], strengthening the plausibility of a gut-derived immunomodulatory route relevant to pollution susceptibility. Because neutrophil activation and extracellular trap biology are increasingly implicated in pollutant responses, dietary exposures that influence SCFA-mediated HDAC activity may be especially relevant to pollutant-triggered neutrophilic airway inflammation [61].

Building on the life-course susceptibility for early-life windows and the peri-natal period, epigenetics acts as a fundamental bridge between dietary exposures and gene expression. An example beyond histone acetylation is that maternal and/or early childhood consumption of unprocessed farm milk has been linked to milk-derived exosome microRNAs acting as additional epigenetic regulators, influencing immune-related gene expression and long-term respiratory resilience [105]. These processes, along with DNA demethylation of the Foxp3 gene with activation of Treg, contribute to the prevention of allergic development and asthma [105]. Conversely, harmful environmental insults like air pollution exploit these same pathways to drive morbidity, reinforcing early life-windows from above [106]; for instance, exposure to polycyclic aromatic hydrocarbons (PAHs) [107,108] and wildfire smoke [109] is associated with Foxp3 hypermethylation, which impairs Treg function and increases IgE levels. Furthermore, pollutants such as diesel exhaust can induce hypermethylation of the IFNG gene, silencing counter-regulatory Th1 responses and skewing immune programming toward a pro-allergic Th2 phenotype [110].

A third axis involves lipid-mediated inflammatory balance and resolution, where higher ω-3 PUFA exposure can promote specialized pro-resolving mediator pathways and reduce arachidonic-acid-derived pro-inflammatory eicosanoids, potentially dampening pollution-triggered airway inflammation [18]. In contrast, diets richer in saturated fat and overall Westernized inflammatory profiles can activate innate immune signaling, including Toll-like receptor (TLR)-related pathways, and are associated with higher FeNO and worse inflammatory profiles in asthma [14], which could increase the magnitude of response to pollutants. Adding to this, in T-cells, as well as ω-3 PUFA such as those found in fish oil, have shown to be related with specific epigenetic regulation through modifications in histone acetylation and T-cell maturation, counteracting through antioxidants defense and determining allergy susceptibility [111].

Overall, the strongest mechanistic consensus to date supports diet as an effect modifier through converging pathways of redox buffering, innate immune priming, including neutrophil recruitment and function, and microbiome-derived immunoregulation. Nonetheless, high-quality causal studies that explicitly test diet-by-pollutant interactions remain a priority [70].

The heterogeneity in supplementation/diet-pollution findings is reflected in the wide range of effect sizes and, in some contexts, contrasting directions of modification. Regarding magnitude, fish oil supplementation has demonstrated up to a 70% attenuation of ozone-induced lung function decrements [19], whereas some antioxidant trials (vitamins C and E) showed negligible or null protective effects on similar endpoints [112]. The direction of effect may also vary by context. For instance, maternal fish consumption was associated with a protective association for infant respiratory symptoms (IRR 0.85) [113], whereas higher maternal vitamin E intake was associated with a 14.29% increase in FeNO in adolescents exposed to PM_2.5_, suggesting a potential synergistic modification [114]. Heterogeneity is further evident across clinical endpoints, as antioxidants often appear more effective at attenuating physiological decrements [115] than at modulating acute neutrophilic inflammation [112,116,117,118]. Finally, population-specific susceptibility makes interpretation difficult, with stronger protective signals reported in children with low baseline antioxidant intake [99] or specific genetic risk alleles [119].

### 5.2. Maternal Diet and Supplementation

Maternal diet during pregnancy may plausibly modulate offspring susceptibility to air pollution by shaping oxidative stress balance and immune programming. However, the available human epidemiologic evidence remains limited and heterogeneous across populations, exposure windows, and outcomes [26,99,114].

In an urban pregnancy cohort (n = 530), higher maternal antioxidant intake, summarized as a composite antioxidant index based on energy-adjusted intakes of β-carotene, vitamins A, C and E, and trace minerals, was associated with lower odds of early-childhood repeated wheeze among Black children (OR 0.37, 95% CI 0.19; 0.73, per IQR increase in composite antioxidant index) [99]. Consistent with the susceptibility framework, the same study suggested that low maternal antioxidant intake identified a subgroup in which late-gestation PM_2.5_ exposure was more strongly associated with repeated wheeze in boys born to Black mothers (33 to 40 gestational weeks: OR 1.74, 95% CI 1.19; 2.54, per 1 μg/m^3^ PM_2.5_).

In a Project Viva analysis using Bayesian kernel machine regression, total vitamin E intake (from foods and supplements) suggested potential effect modification in the opposite direction, consistent with heterogeneity across nutritional exposures and contexts: a “PM_2.5_ * vitamin E” interaction was detected (*p* = 0.02), and among participants in the highest quartile of vitamin E intake, PM_2.5_ was associated with a 14.29% increase in FeNO (95% CI 0.49 to 20.94) [114]. In parallel, an earlier Kraków birth cohort (n = 465) reported that greater maternal fish consumption during pregnancy was associated with a lower incidence of infant respiratory symptoms, including coughing (IRR 0.85, 95% CI 0.79; 0.91), wheezing (IRR 0.97, 95% CI 0.95; 0.99), and difficult breathing (IRR 0.94, 95% CI 0.92; 0.97), which is compatible with a potentially more favorable respiratory profile in settings with prenatal PM_2.5_ exposure, although formal interaction testing was not the primary focus [113].

In contrast, a study examining prenatal O_3_ exposure and maternal oxidative balance scores (OBS) in 661 mother–child pairs did not identify robust associations between prenatal O_3_ and lung function at age eight to nine years in primary models. However, three-way interaction models suggested that higher O_3_ exposure was associated with lower child FEV_1_ among Black women with lower OBS and among White women with higher OBS, although these subgroup analyses were limited by sparse data [26].

A summary of findings about the effects of maternal diet and supplementation on the association between air pollution and respiratory outcomes is presented on Table 1.

### 5.3. Foods and Dietary Patterns

#### 5.3.1. Dietary Inflammatory Potential and Fat Quality

Evidence from experimental and epidemiological studies supports a modulatory role of overall dietary quality and fat composition on pollution-related respiratory responses. Among adults, a study conducted in 2024 showed that individuals with less healthy dietary and lifestyle profiles experienced stronger O_3_-related respiratory effects during sleep exposure [120]. In a panel study of 62 healthy adults, habitual ω-3 intake/erythrocyte ω-3 status (and blood ω-6 levels) modified lag-dependent associations between short-term ambient O_3_ and PM_2.5_ and lung function (FEV_1_/FVC). Associations appeared biphasic: more favorable patterns at lag 0 in higher ω-3 (and lower ω-6 PUFA) groups shifted toward adverse associations at later lags (e.g., lag 3–lag 5), suggesting time-dependent heterogeneity rather than uniform protection [121].

Among pediatric populations, findings from a study including 501 Portuguese school-aged children showed that dietary inflammatory potential modified the association between indoor PM and asthma outcomes [17]. Among children with asthma, exposure to PM_2.5_ was more strongly associated with asthma-related outcomes in those consuming a pro-inflammatory diet (OR 1.44, 95% CI 1.01; 2.21), while PM_10_ exposure showed a similar pattern (OR 1.29, 95% CI 1.03; 1.68) [16]. In line with these findings, within the AsthmaDIET study (n = 135), Brigham balance between ω-6 and ω-3 PUFA modified the respiratory impact of indoor PM [122]. Specifically, higher ω-3 intake was associated with fewer asthma symptoms under PM_2.5_ exposure, whereas higher ω-6 intake amplified symptom frequency, nocturnal symptoms, and rescue medication use.

In Romanian school children, diet components linked to antioxidant potential also modified TRAP–symptom relationships: among children living near heavy traffic, infrequent fruit consumption was associated with higher odds of allergy-like symptoms (OR 4.01, 95% CI 0.93; 17.26) compared with children who frequently consumed fruit (OR 0.67, 95% CI 0.29; 1.54; p-interaction = 0.036) [123]. In the same cohort, traffic exposure combined with frequent milk (OR 2.80, 95% CI 1.24; 6.31) and frequent yogurt consumption (OR 2.86, 95% CI 1.05; 7.75) was associated with higher odds of asthma-like symptoms.

A summary of findings about the effects of food and dietary patterns, including dietary potential and fat quality (this section), Mediterranean-type patterns and plant-based dietary patterns (the following sections) on the association between air pollution and respiratory outcomes is presented on Table 2.

#### 5.3.2. Mediterranean-Type Patterns

The Mediterranean diet, characterized by high intakes of fruits, vegetables, whole grains, and unsaturated fats, has been proposed as a broader anti-inflammatory dietary pattern [11,12] that may buffer pollution-related effects [127]. In a 22-week prospective study of 158 children with asthma and 50 children without asthma, stratified models suggested effect modification by O_3_ among asthmatic children: in the highest O_3_ quartile, higher adherence to Mediterranean-type patterns (Mediterranean Diet Index) was associated with higher FVC (β 0.081 L, 95% CI 0.010; 0.152) [124].

In adults, analysis of 29,032 participants in the WHO Study on Global AGEing and Adult Health showed that habitual fruit and vegetable intake modified the association between long-term ambient PM_2.5_ and lung function, with attenuated PM_2.5_-related decrements among participants reporting higher intake [125]. For each 10 μg/m^3^ increase in PM_2.5_, the reduction in FEV_1_ was larger in the low fruit intake group (β −103.65 mL, 95% CI −168.89; −38.42) than in the high fruit intake group (β −57.96 mL, 95% CI −99.45; −16.48). Similarly, for FEF_25–75_, the decline was stronger under low fruit intake (β −141.40 mL/s, 95% CI −220.16; −62.63) than under high fruit intake, where the estimate was not statistically significant (β −39.38 mL/s, 95% CI −128.27; 49.52). When stratifying by vegetable intake, PM_2.5_-related declines remained significant for FEF_25–75_ in the high vegetable intake group (β −99.54 mL/s, 95% CI −169.60; −29.47), while estimates for FEV_1_ in the high vegetable intake group were not statistically significant.

#### 5.3.3. Plant-Based Dietary Patterns

Evidence regarding plant-based dietary patterns as modifiers of pollution–asthma associations remain limited and inconsistent. In the Nurses’ Health Study II, which examined long-term exposure to PM_2.5_, NO_2_, and O_3_ among women with asthma followed over 17 years, no statistically significant interactions were found between adherence to a plant-based diet index and pollutant exposure in relation to asthma exacerbations [126]. These null interaction findings may reflect limited power for interaction testing and heterogeneity in the quality of plant-based diets, rather than the absence of biological plausibility.

As said in the previous section, you can find a summary of these findings in Table 2.

### 5.4. Nutritional Supplementation

#### 5.4.1. Fatty Acids and Oils

In a randomized trial with controlled O_3_ exposure in healthy young adults, fish oil (FO) and olive oil (OO) supplementation before acute O_3_ exposure modified the magnitude of the O_3_-related lung function decrease [19]. In the control group, O_3_ induced a mean reduction in FEV_1_/FVC of −4.67% (95% CI −7.84; −1.50, n = 12), whereas this decrease was markedly attenuated in the FO group (−1.40%, 95% CI −3.16; 0.36, n = 15) and less consistently in the OO group (−3.06%; 95% CI −4.39; 1.73, n = 16). The between-group comparison showed significant protection with FO for FEV_1_/FVC (*p* = 0.01), corresponding to ~70% attenuation of the control decrement, whereas the FO vs. control contrast was significant but the OO vs. control contrast did not reach statistical significance. Similar patterns were observed for FEV_1_ and FVC, although between-group differences were weaker for these endpoints.

In the Childhood Allergy and Pollution Study, which randomized supplementation from infancy to five years, FO also modified associations between TRAP and allergic/respiratory outcomes [128]. For house dust mite (HDM) sensitization at age five, increasing TRAP exposure was associated with a higher risk of a positive HDM skin prick test in the control group (RR 1.55, 95% CI 1.04; 2.31), but not in the FO group (RR 0.95, 95% CI 0.62; 1.45). For lung function, TRAP exposure was associated with a lower pre-bronchodilator FEV_1_/FVC between ages five and eight among non-movers (participants who did not change residence) in the control group (mean difference −0.03, 95% CI −0.05; −0.01), whereas no corresponding decrease was observed in the FO group (0.00, 95% CI −0.02; 0.02; p-interaction = 0.031).

A summary of findings about the effects of nutrients supplementation, including fatty acids and oils and antioxidant and redox-targeted nutrients (including vitamins C and E, vitamin E isoforms, thiol-based antioxidants) on the association between air pollution and respiratory outcomes is presented on Table 3.

#### 5.4.2. Antioxidant and Redox-Targeted

##### Vitamins C and E

Evidence from children suggests that baseline antioxidant status can modify acute pollution-related functional responses. In children with asthma, low vitamin C intake amplified O_3_-associated reductions in FEF_25–75_, particularly in those with higher oxidative-stress genetic susceptibility [119]. Accordingly, among persistent asthmatics with four to six risk alleles, a 60 ppb O_3_ increase was associated with a larger reduction in FEF_25–75_ in the low vitamin C group (−97.2 mL/s, 95% CI −143.1; −51.4) than in the high vitamin C group (−47.7 mL/s, 95% CI −95.4; 0.0) [119]. Moreover, in children with asthma in a high-pollution setting (Mexico City), vitamins C + E supplementation modified short-term O_3_–lung function associations: in the placebo arm, higher O_3_ on the prior day was associated with lower FEF_25–75_ (β −13.32 mL/s per 10 ppb; *p* < 0.001) and lower FEV_1_ (β −48.0 mL per 10 ppb; *p* = 0.03), whereas these associations were attenuated or absent under supplementation, with the most consistent between-group contrast reported for FEF_25–75_ [129].

In adults, supplementation trials have shown mixed/limited modification of O_3_ effects on spirometry and inflammation. In one crossover trial of O_3_-responsive subjects, vitamin C + E supplementation did not materially modify O_3_-associated lung function declines (FEV_1_ decrease 8.5% on O_3_ after placebo vs. 7.3% after antioxidants), with similar airway neutrophilia across conditions [112]. In addition, a study including a combination of vitamin C, α-tocopherol, and a vegetable cocktail reported modification of the association between O_3_ and lung function, reducing O_3_-associated declines by ~30% for FEV_1_ (*p* = 0.046) and 24% for FVC (*p* = 0.055) vs. placebo, while inflammatory markers did not show consistent modification [116]. By contrast, in an SO_2_ challenge study in adults with asthma, pretreatment with vitamins C and E appeared to attenuate SO_2_-related bronchoconstrictive responses [115]. Specifically, FEV_1_ decline was 12.9% with placebo vs. 5.9% with vitamins, and airway resistance increases were also smaller (57.8% placebo vs. 35.6% with vitamins).

##### Vitamin E Isoforms: γ-Tocopherol

In adults with mild asthma studied in a randomized, double-blind, placebo-controlled crossover design, 14 days of γ-tocopherol (γT) supplementation were associated with improvements in baseline airway biomarkers, including lower sputum eosinophilia (% eosinophils *p* = 0.04; eosinophils per mg sputum *p* = 0.01) and lower mucin outcomes (total mucins *p* = 0.03; MUC5AC *p* < 0.0001) vs. placebo [130]. Following a standardized inhaled endotoxin (LPS) challenge, the placebo period showed a clear neutrophilic response (e.g., at 6 h, increases in % PMNs and PMNs/mg with *p* < 0.01), whereas γT significantly attenuated sputum neutrophil percentages in mixed models at 6 h (*p* = 0.04) and 24 h (*p* = 0.02) post-challenge. Notably, the transient slowing of mucociliary clearance observed after endotoxin during placebo (*p* < 0.01) was not present during γT (*p* = 0.6), supporting an effect on mucociliary/epithelial host defense alongside inflammatory modulation.

In a separate double-blind, placebo-controlled crossover study in adults with mild allergic asthma undergoing controlled O_3_ exposure, γT did not reduce eosinophilic or neutrophilic airway inflammation or alter cytokine profiles, but it attenuated the decline in central mucociliary clearance observed during placebo after O_3_ [117].

On the other hand, in a pilot randomized controlled trial with controlled wood-smoke particle exposure, γT supplementation increased systemic γT/γ-CEHC (confirming uptake) and did not reduce the neutrophilic response to wood smoke (primary endpoint), but it was reported to prevent wood-smoke-associated increases in sputum eosinophils among participants with detectable eosinophils [118].

Overall, across controlled exposure models (O_3_, wood smoke, and LPS challenge), γT signals appear more consistent for mucociliary defense and eosinophilic/allergic pathways than for acute neutrophilic inflammation, although evidence remains limited and endpoint specific.

##### Thiol-Based Antioxidants: N-Acetylcysteine (NAC)

N-acetylcysteine (NAC) is described as a thiol-based agent with a long-standing mucoactive (mucolytic) role, supporting airway clearance by decreasing sputum viscosity [131]. It has also been emphasized that NAC should not be framed solely as a mucolytic, given pleiotropic actions relevant to airway disease, including antioxidant and anti-inflammatory effects in the airways [132]. More recently, NAC’s established clinical use has been summarized, including its role as a mucolytic and its broader antioxidant and anti-inflammatory profile, noting that its anti-inflammatory effects may relate to modulation of pathways such as NF-κB and reductions in cytokine signaling in some contexts [133].

In a randomized, double-blind, crossover study, 26 non-smokers took NAC 600 mg three times daily for 6 days and then underwent a 2 h diesel exhaust exposure (300 μg/m^3^ PM_2.5_) (vs. filtered air, depending on condition) [134]. Among hyperresponsive individuals, NAC reduced baseline airway responsiveness by ~20% (*p* = 0.001), while under placebo, diesel exhaust increased airway responsiveness by ~42% vs. filtered air (*p* = 0.03).

As said in the previous section, you can find a summary of these findings in Table 3.

#### 5.4.3. NRF2 Activation: Sulforaphane/Broccoli Sprouts

Evidence from controlled air-pollution exposure models in humans is mixed and appears sensitive to formulation, dose standardization, and endpoint selection. In a clinical diesel exhaust particle (DEP) challenge model, an aqueous intranasal DEP suspension (300 μg) produced a robust nasal inflammatory response [100]. In the same participants, total nasal white blood cell counts (WBC) increased by 66% vs. screening and by 85% vs. control levels at 24 h post-DEP. When the DEP challenge was preceded by four days of a standardized broccoli sprout extract (BSE) delivering 100 μmol sulforaphane (SFN)/day, the total nasal lavage cell count decreased by 54% compared with DEP challenge without BSE (*p* < 0.001).

However, this anti-inflammatory signal has not been consistently reproduced across airway compartments and inflammatory phenotypes. In a proof-of-concept O_3_ exposure model in healthy adults, SFN supplementation via broccoli sprout homogenate (BSH) did not induce antioxidant gene expression and did not protect against O_3_-induced neutrophilic airway inflammation, despite evidence of systemic SFN exposure [135]. Similarly, in a double-blind randomized trial in 40 adults with atopic asthma, three days of 100 g/day whole broccoli sprouts (vs. 100 g/day alfalfa sprouts) did not reduce FeNO (primary outcome), did not induce cytoprotective antioxidant genes in PBMCs or nasal epithelial cells, and did not improve lung function or inflammatory/oxidative stress biomarkers, despite increased serum SFN conjugates [136].

Beyond airway inflammation endpoints, in a 12-week randomized trial in 291 adults in Qidong, China, a broccoli sprout beverage providing 600 μmol glucoraphanin + 40 μmol SFN daily led to rapid and sustained increases in urinary excretion of glutathione-derived conjugates of benzene (61%) and acrolein (23%) vs. placebo (*p* ≤ 0.01) [137].

Together, these findings suggest that broccoli sprout-derived interventions can enhance systemic detoxication biomarkers, but evidence for consistent protection against pollutant-induced airway inflammation or asthma-relevant endpoints remains limited and endpoint-dependent.

A summary of findings about the effects of nutrient supplementation, including NRF2 activation: sulforaphane/broccoli sprouts and vitamin D on the association between air pollution and respiratory outcomes is presented on Table 4.

#### 5.4.4. Vitamin D

Randomized evidence in pediatric asthma does not support routine vitamin D supplementation as an exacerbation-prevention strategy in unselected children with low vitamin D. In the Vitamin D Kids Asthma (VDKA) randomized, double-blind, placebo-controlled trial, children aged six to 16 years with persistent asthma receiving inhaled corticosteroids and baseline 25(OH)D < 30 ng/mL were assigned to vitamin D_3_ (4000 IU/day) or placebo for 48 weeks [139]. The trial was stopped early due to futility, while supplementation did not improve time to first severe exacerbation, with mean time to first exacerbation of 240 days in the vitamin D_3_ arm versus 253 days in the placebo arm (adjusted HR 1.13; 95% CI 0.69; 1.85), and no consistent improvements across key secondary outcomes.

A post hoc analysis of VDKA evaluated long-term residential PM_2.5_ exposure as an effect modifier and reported that the effect of vitamin D supplementation on severe exacerbations differed by PM_2.5_ exposure, with patterns consistent with greater benefit among children living in higher PM_2.5_ environments and little or no benefit in lower-exposure settings [138]. While this observation aligns with the hypothesis that correcting deficiency may be most clinically relevant in high-pollution contexts, it remains hypothesis-generating given the post hoc design, potential exposure misclassification, and limited generalizability across regions with different pollutant mixtures and indoor co-exposures [138].

A summary of these findings is presented in Table 4.

## 6. Strategies, Recommendations and Policy Implications

### 6.1. Overarching Principles: “Reduce Exposure at Source”, Protect High-Risk Groups, and Avoid Widening Inequities

Across the life course, the most effective strategy to reduce asthma morbidity attributable to air pollution is primary prevention through emissions reduction, complemented by targeted protection of susceptible groups, including people with asthma, pregnant people, infants and children, older adults, and individuals with cardiopulmonary comorbidities. The WHO Global Air Quality Guidelines (AQGs) provide health-based benchmark concentrations for major pollutants, such as PM_2.5_ annual mean (5 µg/m^3^; 24 h 15 µg/m^3^; NO_2_ annual mean 10 µg/m^3^; O_3_ peak season 60 µg/m^3^) and interim targets to guide staged implementation [140] (detailed in Supplementary Table S1).

At the European policy level, the recast Ambient Air Quality Directive (EU) 2024/2881 updates binding standards and governance mechanisms, including air quality plans, monitoring, and enforcement provisions, aiming for substantial reductions by 2030 and a longer-term “zero pollution” trajectory” [141].

However, benchmarking against the 2021 WHO AQGs continues to show that exceedances remain widespread, reinforcing the need for accelerated action and cross-sector coordination, including transport, energy, housing, industry, and agriculture [140].

A key implication of the evidence reviewed in Section 2, Section 3 and Section 4 is that pollution-related asthma risk is heterogeneous: air pollution interacts with critical developmental windows and co-existing susceptibility factors, including socioeconomic disadvantage (environmental inequality), psychosocial stress, obesity and metabolic dysfunction, indoor co-exposures, aeroallergens, and diet. Therefore, mitigation should be framed as multi-level and life-course sensitive, prioritizing high-impact interventions while ensuring equitable access to protective measures and avoiding the unintended shift in responsibility to individuals for structurally driven exposures.

### 6.2. Policy- and Community-Level Prevention: Structural Levers That Shift Population Risk

Because air pollution is a population-level hazard, the most impactful interventions are those that shift the exposure distribution upstream, rather than relying primarily on individual avoidance behaviors. Three policy domains map directly onto the exposure contexts emphasized in this review: (i) outdoor emissions, (ii) indoor air quality governance, and (iii) environmental justice and accountability.

(i) Ambient emissions reduction in transport, industry, and urban planning:

Policies that reduce TRAP, including low-emission zones, fleet electrification, cleaner public transport, and safe walking/cycling networks can reduce asthma morbidity at scale while delivering co-benefits, such as reduced noise, climate mitigation, and opportunities for physical activity. This is consistent with evidence that policy-driven air quality improvements translate into measurable respiratory benefits, including improved lung development trajectories [47,48]. Implementation should prioritize hotspots and high-vulnerability neighborhoods, because equal absolute reductions can still leave substantial relative disparities if baseline exposures and vulnerabilities are unequally distributed (environmental inequality framing) [68,69,70,71].

(ii) Indoor air quality governance (homes/schools): dampness/mold, cleaning products/VOCs, and tobacco exposure:

Given the proportion of time spent indoors, particularly in early life, indoor environments are a major leverage point. Recent EAACI guidance synthesizes evidence linking indoor pollutants, including dampness/mold, cleaning agents, VOCs, and pesticides with new-onset asthma and asthma-related outcomes, emphasizing the indoor exposome and pollutant synergies [9]. Policy actions consistent with this review include:

- Housing quality standards and enforcement to prevent and remediate dampness/mold, including minimum ventilation requirements moisture control, and accountability mechanisms in rental housing.
- Stronger regulation and transparency for cleaning/consumer products, including ingredient disclosure and restrictions on high-irritant VOCs/fragrances, paired with guidance for schools and workplaces to minimize respiratory irritants [9].
- Smoke-free environments, including multi-unit housing protections and enforcement to reduce children’s exposure to secondhand smoke and emerging nicotine/aerosol sources.

(iii) Environmental justice, risk communication, and accountability.

Air-quality alert systems and the integration of the Air Quality Index into public messaging can support short-term exposure reduction during peaks, particularly when linked to actionable guidance and clinical self-management. However, evidence suggests effects are variable and depend on implementation, including access, literacy, feasibility of behavioral change, and trust [142]. Alerts should therefore be framed as supportive tools, not substitutes for emissions reduction, and designed to reach high-risk communities (language, digital access, and practical options).

Lastly, accountability mechanisms, such as robust monitoring, enforcement, and equity metrics embedded in air quality plans are essential to ensure that improvements reach communities experiencing environmental injustice [68,69,70,71].

### 6.3. Clinical Integration: “Pollution-Aware Asthma Care”

A practical goal is to incorporate pollution and its modifiers into asthma care in ways that are actionable, equitable, and consistent with established management frameworks. GINA emphasizes written asthma action plans, controller adherence, and trigger management as part of routine care [143]. Building on that foundation and the modifiers discussed in this review, “pollution-aware asthma care” can be operationalized through three elements:

- Risk stratification beyond pollutants alone: identify patients with cumulative vulnerability, such as pregnancy, early childhood, severe/poorly controlled asthma, obesity/metabolic comorbidity, psychosocial stress, smoke exposure, damp housing, high-allergen environments, and socioeconomic constraints [62,69,70,71,72,73,74,75,76,144].
- Action plans that incorporate exposure information: where feasible, link symptom monitoring and medication steps with air-quality alerts, focusing on realistic behavioral adaptations (e.g., timing/location of outdoor activity during peaks, school-day planning, and household practices that reduce indoor pollutant accumulation) [142,143]
- Address modifiable co-exposures and treatable traits: systematically assess household smoke exposure, dampness/mold, irritant cleaning products, and allergen interactions; optimize guideline-based pharmacologic management; and address treatable traits such as obesity and diet quality that may amplify pollution susceptibility [9,62].

This approach aligns with evidence that pollution-related asthma risk reflects interactions between pollutants and modifiable/non-modifiable host and contextual factors, requiring coordinated policy and clinical levers rather than single-component interventions [145].

### 6.4. Integrated Strategies

All integrated strategies are detailed in Supplementary Table S1.

#### 6.4.1. Environmental Inequality and Socioeconomic Vulnerability

Policy: (1) prioritize air quality and housing interventions in high-burden neighborhoods; (2) embed equity metrics in air-quality plans; (3) and fund remediation, including housing dampness, school ventilation where households cannot self-finance exposure reduction [68,69,70,71].

Health systems: reduce barriers to guideline-based asthma care, including medication affordability, continuity of care, school-based asthma programs, community health workers, because differential access sustains disparities even when ambient air improves [76].

#### 6.4.2. Psychosocial Stress and Chronic Adversity

Evidence supports synergistic effects of stress and pollution on asthma morbidity and lung function via shared oxidative/inflammatory pathways, but intervention evidence remains limited [145].

Clinical and community: screen for psychosocial stressors and connect families to support services; consider behavioral interventions as part of comprehensive asthma care, particularly in high-exposure settings.

Policy: evaluate “co-benefit” policies, such as, housing stability, social protection, using respiratory outcomes as part of impact assessment.

#### 6.4.3. Obesity and Metabolic Dysfunction (Treatable Trait Approach)

Obesity is a treatable trait associated with poorer asthma control and may amplify vulnerability to air pollutants [62,77,78].

Clinical: incorporate weight and metabolic assessment into asthma reviews; deliver structured support for sustainable dietary patterns and activity compatible with asthma control [62].

Policy: strengthen healthy school food environments and affordability of minimally processed foods, addressing both obesity prevalence and dietary inflammatory potential [16,17,62].

#### 6.4.4. Indoor Co-Exposures

Tobacco/nicotine aerosols: enforce smoke-free environments around children; support cessation; prioritize protections in multi-unit housing [9].

Cleaning/consumer products: promote practices that minimize irritant exposure, including avoid cleaning during children’s presence, ensure ventilation during and/or after use, avoid product mixing, use diluted concentrations where appropriate, aligned with EAACI’s indoor pollutant guidance [9].

Dampness/mold: implement remediation pathways (moisture control, leak repair, ventilation), with clear referral routes for vulnerable households.

#### 6.4.5. Aeroallergens as Co-Triggers

Concurrent exposure to aeroallergens and air pollutants can potentiate airway inflammation and exacerbate asthma, supporting integrated advice that accounts for both pollutant peaks and allergen seasons in sensitized patients [9,51].

#### 6.4.6. Diet and Supplementation: Translating Mechanistic Plausibility into Pragmatic Recommendations

This review supports diet as a plausible susceptibility modifier of pollution-related airway injury through redox buffering, innate immune priming, and microbiome–immune crosstalk [61]. However, evidence is heterogeneous across pollutants, settings, and endpoints, and the strongest signals are not uniform across supplements or populations (detailed in Supplementary Table S1). A guideline-consistent, evidence-aligned position is therefore:

- Prefer “food-first” resilience-building dietary patterns: Prioritize dietary patterns with anti-inflammatory and antioxidant profiles with higher intake of fruits, vegetables, legumes, whole grains, and healthy fats, such as fatty fish and unsaturated fats, consistent with observational evidence suggesting attenuation of pollution-associated respiratory decrements in higher-quality dietary contexts [14,17,125]. Conversely, reducing ultra-processed foods and pro-inflammatory dietary profiles is relevant, given the links to worse asthma outcomes and the demonstrated interaction between dietary inflammatory potential and indoor PM exposure in children [16,17].
- Focus on fat quality and fiber as mechanistically coherent targets: Given evidence that ω-3/ω-6 PUFA balance may modify pollution-related asthma symptoms and lung function responses [122], dietary counseling can explicitly encourage regular ω-3 PUFA sources and moderation of dietary patterns dominated by ω-6-rich processed fats. In parallel, improving fermentable fiber intake is mechanistically coherent with microbiome-derived immune regulation pathways implicated in pollutant-triggered inflammation [61,103,104].
- Use supplements cautiously and selectively: Controlled exposure and supplementation trials indicate that certain supplements (e.g., fish oil; antioxidant combinations in specific high-pollution pediatric contexts) may attenuate acute pollutant-induced functional or inflammatory changes [19,124]. However, consistency across asthma-relevant clinical outcomes is limited, and population-wide supplementation is not justified as a primary mitigation strategy. Where supplementation is considered, a pragmatic approach is to: (a) prioritize correction of clear dietary inadequacy/deficiency; (b) consider baseline status, dose, and context; and (c) avoid portraying supplements as substitutes for emission reduction or guideline-based asthma pharmacotherapy.

Overall, diet should be framed as one component of an integrated mitigation package, alongside smoke exposure reduction, indoor air quality improvements, obesity management, and stress-informed care, reflecting asthma’s multifactorial etiology and the interaction between exposures and treatable traits [62,143].

### 6.5. Key Gaps and Implementation Priorities

Several gaps currently limit the translation of pollution–modifier evidence into practice and policy:

- Intervention evidence: more rigorous evaluations of exposure-reduction strategies on asthma outcomes are needed, particularly outside high-income settings and across combined indoor–outdoor exposure scenarios [142].
- Diet-by-pollution causality: high-quality trials and quasi-experimental studies explicitly testing diet/pattern interventions as effect modifiers of pollution-related asthma outcomes remain scarce (beyond nutrient supplementation paradigms).
- Equity and feasibility: many “individual-level” actions, such as behavior change and home remediation, are not equally feasible; evaluations should include equity endpoints and avoid shifting responsibility to those least able to act [68,69,70,71].
- Mixtures and co-exposures: research and policy should move beyond single-pollutant frameworks to reflect realistic mixtures and the indoor exposome, consistent with EAACI’s framing [9].
- Precision prevention without delay: clarify which subgroups benefit the most from targeted mitigation (e.g., early-life windows, obesity/metabolic dysfunction, psychosocial stress, poor diet quality), while ensuring subgroup identification does not delay population-wide emissions reduction.

## 7. Conclusions

This narrative review showed that both outdoor and indoor pollutants contribute to asthma development and exacerbations and are associated with impaired lung function. Susceptibility is not uniform: stronger adverse associations are repeatedly reported during pregnancy and childhood and among populations facing higher exposure burdens and vulnerability, including those with socioeconomic disadvantage, chronic stress, obesity and metabolic dysfunction, and relevant co-exposures, including aeroallergens and indoor mixtures.

We also found converging mechanistic support that diet may influence pathways relevant to pollution-related respiratory harm, particularly oxidative stress, epithelial-immune responses, inflammatory lipid balance, and microbiome-related immune modulation. However, human evidence remains mixed for specific nutrients and supplementation, while higher overall diet quality shows the most consistent signal of potential protection. We conclude that pollution control remains central, and that improving diet quality is a plausible, low-risk adjunct strategy rather than a substitute for emission reduction and guideline-based asthma care.

## Figures and Tables

**Table 1:** Effects of maternal diet and supplementation on the association between prenatal air pollution exposure and respiratory outcomes in children. Studies are found according to their mentioning order within chapter.

Studies	Participants and Design	Intervention/ Exposure	Outcomes	Findings
Chiu (2022) [99]	Prospective birth cohort 530 mother–child pairs PRISM, Boston and New York, USA; Daily prenatal PM_2.5_	Block98 FFQ, AI	Respiratory symptoms Repeated wheeze (≥2 episodes) at ~4 years	AI was significantly higher with decreased wheeze in black children.PM_2.5_ increases wheeze significantly among boys born to Black mothers with low AI (at 33–40 weeks gestation). Associations between prenatal PM_2.5_ exposure and childhood wheeze were modified by maternal antioxidant intake, race/ethnicity, and child sex.
Dearborn (2025) [26]	Prospective birth cohort 661 mother–child pairs CANDLE cohort, USA Pre-natal O_3_ exposure	Diet, reflected in OBS	Lung function at 8–9 years FEV_1_, FVC, FEV_1_/FVC, FEF_25–75_	Three-way interaction models, higher O_3_ was associated with lower child FEV_1_ among black women with lower OBS and among white women with higher OBS. No significant association between prenatal O_3_ exposure and lung function. No effect modification by OBS or maternal race was found in 2-way models.
Sordillo (2018) [114]	Prospective pre-birth cohort 857 mother–child pairs Project Viva, a Massachusetts Outdoor PM_2.5_,	Semi-quantitative FFQs; folates, prenatal vit. D, E, ω-3 PUFA supplementation	Airway inflammation at ~12 FeNO, and Total IgE	Significant and synergistic interaction between prenatal vit. E and PM_2.5_, increasing FeNO. In the highest quartile of PM_2.5_ exposure, vit. E was associated with 8.42% increase in FeNO. In the highest quartile of vitamin E, PM_2.5_ was associated with a 14.29% increase in FeNO. Higher prenatal vit D and ω-3 PUFA decreased FeNO significantly and folates non significantly.
Jedrychowski (2008) [113]	Prospective birth cohort 465 newborns (0–2 years old) Krakow, Poland. PM_2.5_ at the 2nd trimester	Maternal fish consumption	Respiratory symptoms (coughing, wheezing, difficult/puffy breathing in first 2 years)	Fish consumption during pregnancy was significantly protective, reducing risk of coughing, wheezing, and breathing difficult. Higher PM_2.5_ significantly increases coughing, wheezing, and difficult breathing.

Abbreviations: AI, antioxidant index; FeNO, fractional exhaled nitric oxide; FEF_25–75_, forced expiratory flow between 25% and 75% of forced vital capacity; FEV_1_, forced expiratory volume in 1 s; FEV_1_/FVC, ratio of forced expiratory volume in 1 s to forced vital capacity; FVC, forced vital capacity; FFQ, food-frequency questionnaire; OBS, oxidative balance score; PM_2.5_, particulate matter with aerodynamic diameter ≤ 2.5 μm; PM_10_, particulate matter with aerodynamic diameter ≤ 10 μm; PRISM, Programming of Intergenerational Stress Mechanisms; PUFA, polyunsaturated fatty acids.

**Table 2:** Effects of food and dietary patterns, including dietary potential and fat quality (this section), Mediterranean-type patterns and plant-based dietary patterns (the following sections) on the association between air pollution and respiratory outcomes. Studies are found according to their mentioning order within the chapter.

Studies	Participants and Design	Intervention/ Exposure	Outcomes	Findings
Li (2024) [120]	Prospective observational cohort 81 adults; Beijing, China. Real-time Indoor O_3_ (sleep)	Baseline lifestyle, Diet	Lung function, FVC, FEF_25–75_; Airway inflammation	Indoor O_3_ exposure during sleep was associated with less airway function, but not with airway inflammation. Stronger effects are noted among those with worse dietary patterns and specific lifestyles.
Tong (2022) [121]	Prospective panel study 62 healthy adults North Carolina, USA Short term ambient O_3_ PM_2.5_	ω-3 PUFA groups, high or low intake	Lung Function FVC, FEV_1_	FVC was positively associated with O_3_ at lag0 in the high ω-3 PUFA whereas it was null in the low ω-3 PUFA group. The association shifted to being negative at lag4 for high ω-3 PUFA and remaining low for low ω-3 PUFA.
de Castro Mendes (2020) [17]	Cross-sectional 501 children (7–12 years) 20 public schools Porto, Portugal, Indoor PM_2.5_, PM_10_, UFP, CO_2_, O_3_ NO_2_	DII—questionnaire answered by children, without parents (24 h recall)	Lung function, FEV_1_ Airway inflammation, FeNO Atopic status; Respiratory symptoms	In children within asthma treatment, PM_2.5_ exposure effect 44% higher significantly with pro-inflammatory diets, while lower significantly with anti-inflammatory diets. Children with severe asthma and under asthma treatment, PM_10_ exposure effect was 25% and 30% higher significantly, respectively, for pro-inflammatory diets. PM_2.5_ levels and DII for lung function were not associated significantly.
Brigham (2019) [122]	Prospective longitudinal observational; 135 children (5–12 years) AsthmaDIET Study; Baltimore, Maryland, USA; PM_2.5_, PM_10_ home weekly concentrations	FFQ with 7-day recall, ω-3 and ω-6 fatty acid	PM-related asthma symptoms per day and night Albuterol use	Higher ω-3 PUFA intake reduced effect of indoor PM_2.5_ on symptoms. Higher ω-6 PUFA intake increased effect of indoor PM_2.5_ on symptoms and neutrophil %. Intake of ω-6 PUFA increased significantly PM_2.5_ effect on albuterol use and nocturnal symptoms and PM_10_ increased significantly nocturnal symptoms. No significant association was noted between ω-6 PUFA nor ω-3 PUFA in what comes to PM_2.5_ or PM_10_ effect on % eosinophils.
Lawrence (2020) [123]	Cross-sectional observational study 280 school children SINPHONIE project Romania Household and residential environmental exposures, TRAP	Self-reported dietary habits	Self-reported health symptoms (allergy-like, asthma-like, flu-like)	Frequency of fruit consumption significantly interacted with living near heavy traffic on allergy-like symptoms. Although no significant association between fruit frequency and allergy-like symptoms, those with less fruit consumption living near heavy traffic had higher odds of asthma-like and flu-like symptoms. Asthma-like symptoms increased significantly with low vegetable consumption or if living near heavy traffic and frequent consumption of dairy products (e.g., milk or yogurt).
Romieu (2009) [124]	Prospective longitudinal cohort 158 asthmatic and 50 non-asthmatics Children; Children’s Hospital of Mexico, Mexico; PM_2.5_, O_3_ NO_2_ 22 weeks	Dietary intake—108-item FFQ, FVI and MDI	Lung function, FVC, FEV_1_; Airway inflammation, nasal lavage, FeNO	Significant positive interaction between FVI and O_3_ level for both FEV_1_ and FVC and MDI and O_3_ level for FVC. Higher MDI children had higher FEV_1_ and FVC. Higher FVI was significantly, inversely associated with IL-8 levels in nasal lavage. No effect of diet was observed among non-asthmatic children.
Lin (2018) [125]	Cross-sectional, population-based 29,032 adults (≥50 years); WHO Study on global AGEing, adult health; Annual mean PM_2.5_ satellite data	Self-reported dietary fruit and vegetables	Lung function FVC, FEV_1,_ FEV_1_/FVC, PEF, FEF_25–75_	Higher ambient PM_2.5_ exposure was significantly associated with reduced lung function Lower effect estimates were observed among those with higher consumption of fruit and vegetables.
Wang (2025) [126]	Prospective longitudinal cohort 4326 women with asthma Asthmatic Women Nurses’ Health Study II, USA; Residential ambient PM_2.5_, NO_2_ and O_3_—48 months	PDI scores derived from FFQ	Asthma exacerbations in the past year (1998 and 2014)	In single pollutant-models, long-term exposure to ambient NO_2_ and PM_2.5_ even at low levels, may increase asthma exacerbation risk in women, but is not attenuated by a plant-based diet as measured herein. In multi-pollutant models, NO_2_ exposure remained significantly associated with asthma exacerbation risk. Pollutants and PDI score on asthma exacerbations had no significant association.

Abbreviations: DII, diet inflammatory index; FeNO, fractional exhaled nitric oxide; FEF_25–75_, forced expiratory flow between 25% and 75% of forced vital capacity; FEV_1_, forced expiratory volume in 1 s; FEV_1_/FVC, ratio of forced expiratory volume in 1 s to forced vital capacity; FVC, forced vital capacity; FFQ, food-frequency questionnaire; MDI, mediterranean diet index; OBS, oxidative balance score; NO_2_, nitric dioxide; O_3_, ozone; PDI, plant-diet index; PM, particulate matter; PM_2.5_, particulate matter with aerodynamic diameter ≤ 2.5 μm; PM_10_, particulate matter with aerodynamic diameter ≤ 10 μm; PUFA, polyunsaturated fatty acids; ROS, reactive oxygen species; TRAP, traffic-related air pollution.

**Table 3:** Effects of nutrient supplementation, including fatty acids and oils and antioxidant and redox-targeted nutrients (including vitamin C and E, vitamin E isoforms, thiol-based antioxidants) on the association between air pollution and respiratory outcomes. Studies are found according to their mentioning order within the chapter.

Studies	Participants and Design	Intervention/ Exposure	Outcomes	Findings
Chen (2022) [19]	Randomized controlled trial 43 healthy participants Research Triangle Area of Central North Carolina, USA; O_3_ chamber	3 g/day of FOS, 3 g/day OO, or CTL four weeks	Lung function, FVC, FEV_1_, FEV_1_/FVC, FVC; Airway inflammation sputum neutrophil%	FEV_1_ and FEV_1_/FVC higher significantly in FO group vs. CTL immediately post-O_3_. OO higher non significantly FEV_1_. FO blunted significantly O_3_-induced loss of FEV_1_/FVC (70%) and non-significantly ameliorated FVC (19%) and FEV_1_ (48%) reductions. OO non-significant 34% protection against O_3_-induced loss in FEV_1_/FVC. No significant differences in sputum neutrophil % post O_3_ exposure.
Hansell (2018) [128]	Randomized, placebo-controlled birth cohort; 400 children CAPS, Sydney, Australia TRAP: HDM (w/IL5 response), SPT	FOS (500 mg of tuna fish oil) or placebo	Lung function FEV_1_/FVC	Significant interactions between FOS and TRAP exposure were observed for HDM SPT, Inhalant SPT, All-allergen SPT, HDM-specific IL-5 response at age 5. Higher TRAP associated with HDM SPT with RR 1.74 for the control group vs. 1.03 for FOS. Mostly to those who did not change residence between 5 and 8 years. In this sub-group, FOS decreased the effect of TRAP on pre-bronchodilator FEV_1_/FVC ratio.
Moreno-Macías (2013) [119]	Prospective panel study 257 asthmatic children Mexico City, Mexico Ambient O_3_	Dietary intake of vit. C, high or low	Lung function FEF_25–75_	The change in FEF_25–75_ per interquartile range (60 ppb) of O_3_ in persistent asthmatic children with low vit. C intake and GSTM1 null was borderline significant, −91.2 mL/s (*p* = 0.06). Persistent asthmatic children with 4 to 6 risk alleles and low vit. C intake showed an average but significant decrement in FEF_25–75_ of 97.2 mL/s per 60 ppb of O_3_. No differential ozone effect by vit. C intake.
Romieu (2002) [129]	Randomized trial, double-blinded 158 asthmatic children Mexico City, Mexico NO_2_, SO_2_, PM_10_, ambient O_3_, RH, T	vit. E; C (50 mg/day; 250 mg/day) or placebo (2 years); FFQ-Scorenut-nutrient	Lung function FEF_25–75,_ FEV_1_, PEF	In those with moderate/severe asthma, O_3_ levels 1 day before spirometry was inversely associated significantly with FEF_25–75_, FEV_1_ and PEF in the placebo, but no significant associations between O_3_ and lung function. Significant attenuation O_3_-related decrements for: FEF_25–75_, PEF. Supplementation weakened significantly the negative correlation of NO_2_ with FEF_25–75_ and FEV_1_. In PM_10_ decrements after highest PM_10_ synergistically with O_3_, modulation of supplementation was non significantly greater.
Mudway (2006) [112]	Randomized, double-blind, placebo-controlled crossover 14 subjects O_3_ sensitive Sweden; O_3_ chamber	Supplementation with vit. C (500 mg/day) and E (100 mg/day) or placebo—7 days	Lung function FEV_1_, FVC, Airway inflammation neutrophilia, total DCC	No protection from vitamin supplementation (−8.5%) versus placebo (−7.3%). O_3_-induced neutrophilia was of a similar magnitude after both treatments. (*p* < 0.05). O_3_ exposure accompanied by a significant decrease in macrophage numbers compared to air. Only BAL IL-6 increased significantly after O_3_ in both placebo and vitamin groups vs. air.
Samet (2001) [116]	Double-blind, randomized controlled dietary intervention 31 healthy adults (18–35 years) USA; O_3_ chamber	250 mg of vit. C, 50 IU of α-tocopherol, 12 oz of vegetable cocktail/day or placebo—15 days	Lung function, FEV_1_, FVC; Airway inflammation, % neutrophils, IL-6, respiratory symptoms	O_3_-induced reductions in FEV_1_ and FVC were 30% and 24% smaller, respectively, in the supplemented cohort. There were no differences between the placebo and supplemented groups in responsiveness of pulmonary function parameters to air exposure. No difference in the percent neutrophils and the concentration of IL-6 recovered in the BAL fluid at 1 h after O_3_ exposure was not different.
Trenga (2001) [115]	Randomized, double-blind, placebo-controlled crossover; 17 asthmatic adults; Washington, USA O_3_ chamber	Dietary antioxidants (400 IU vitamin E/500 mg vitamin C)	Lung function FEF_25–75,_ FEV_1_, PEF Bronchial hyperresponsiveness (10 min-SO_2_)	If given dietary antioxidants responded less severely to sulphur dioxide challenge than those given a placebo (FEV_1_, PEF and mid-FEF). Protective effects were stronger in participants with higher baseline responsiveness to SO_2_. The results suggest that dietary supplementation with vitamins E and C benefits asthmatic adults who are exposed to air pollutants.
Burbank (2018) [130]	Randomized, double-blind, placebo-controlled, crossover 15 adults with mild asthma USA; Inhaled LPS challenge	γT supplementation (1200 mg/day) or placebo for 14 days	Airway inflammation eosinophilic, neutrophilic, sputum mucins (total and 5AC) whole lung and regional MCC	Compared with placebo, γT supplementation significantly reduced pre-LPS sputum eosinophils, reduced baseline sputum mucins, including mucin 5AC, attenuated LPS-induced airway neutrophil recruitment at 6- and 24 h post-challenge. MCC was slowed four hours after LPS challenge in the placebo group, but not in the γT-treated group. Total sputum mucins were reduced (but not mucin 5AC) at 24 h post-LPS challenge during γT treatment compared with placebo.
Burbank (2020) [117]	Randomized, double blind, controlled crossover 15 adults with mild asthma USA; O_3_ chamber	Two γT-enriched gel tabs, 600 mg of γT each or placebo for two days	Airway inflammation eosinophilic, neutrophilic; whole lung and regional MCC	γT supplementation did not significantly reduce pre-O_3_ eosinophilic inflammation or attenuate O_3_-induced neutrophilic airway inflammation compared with placebo. No relevant changes in sputum inflammatory cytokines. Regarding MCC, no O_3_-related changes were detected in either treatment; however, the decrease in central lung clearance seen after O_3_ was only observed during placebo.
Peden (2023) [118]	Randomized, placebo-controlled 11 volunteers sensitive to WSP USA; WSP	Short-course γ-T–enriched supplementation or placebo	Airway inflammation Neutrophilic and eosinophilic	Short-term γ-tocopherol supplementation did not reduce wood smoke–induced neutrophilic airway inflammation. γ-tocopherol prevented wood smoke–induced eosinophilic airway inflammation.

Abbreviations; CTL, control/no supplementation; DCC, differential cell counts; FEF_25–75_, forced expiratory flow between 25% and 75% of forced vital capacity; FEV_1_, forced expiratory volume in 1 s; FEV_1_/FVC, ratio of forced expiratory volume in 1 s to forced vital capacity; FVC, forced vital capacity; FOS, fish oil supplementation; HDM, house dust mite; IU, international unit; LPS, lipopolysaccharide; MCC, mucociliary clearance; OO, olive oil; oz, fluid ounce; PM, particulate matter; PM_2.5_, particulate matter with aerodynamic diameter ≤ 2.5 μm; PM_10_, particulate matter with aerodynamic diameter ≤ 10 μm; RH, relative humidity; T, temperature; TRAP, traffic-related air pollution; SO_2_, sulfur dioxide; SPT, skin prick tests; vit., vitamin; WSP, wood smoke particles; γ-T, γ-tocopherol.

**Table 4:** Effects the effects of nutrient supplementation, including fatty acids and oils and antioxidant and redox-targeted nutrients (including NRF2 activation: sulforaphane/broccoli sprouts and vitamin D) on the association between air pollution and respiratory outcomes. Studies are found according to their mentioning order within the chapter.

Studies	Participants and Design	Intervention/ Exposure	Outcomes	Findings
Carlsten (2014) [134]	Randomized, double-blind, cro- ssover; 26 non-smokers (19–46 years); Vancouver, Canada FA + placebo and DE + PM_2.5_	N-acetylcysteine (600 mg) or placebo capsules three times/day for 6 days	DRS to methacholine Self-reported symptoms	In hyper-responsive adults, antioxidants reduced baseline airway responsiveness and self-reported SABA use significantly. Also, significantly abolished the effect of DE vs. FA on airway responsiveness. FEV_1_ and symptoms were unchanged. A total of 30 h after exposure, non-significantly, antioxidants attenuated the increase in DE vs. FA in hyper-responsive increase in bronchial epithelial cells and neutrophils.
Heber (2014) [100]	Double-blind, crossover 29 healthy non-smoker adults Maryland, Baltimore, USA DEP indoor intranasal challenge	BSE (100 μmol SFN in mango juice) for 4 days	WBC in nasal lavage	Intranasal DEP challenge increased nasal WBC by 66–85%, indicating significant inflammation. Total cell counts decreased significantly by 54% when DEP challenge was preceded by daily BSE administration for 4 days.
Duran (2016) [135]	Randomized, placebo, controlled crossover; 15 healthy adults (18–50 years); North Carolina, USA; O_3_ chamber	Randomized (1:1 ratio) to consume either 200 g of BSH/ASH, which lacks SFN	Airway inflammation % PMNs in sputum, nasal epithelial cells NRF2 related	Supplementation of SFN with broccoli sprout homogenate in healthy human subjects did not induce expression of antioxidant genes or protect against neutrophilic airway inflammation in an O_3_-exposure model.
Sudini (2016) [136]	Randomized, double-blind, placebo-controlled clinical trial 40 asthmatic adults (18–50 years); Baltimore, USA	Dietary intervention with BSH/ASH (source of SFN) 100 g/day for 3 days	Lung function, FEV_1_ Airway inflammation FeNO, PBMCs and NEC Respiratory symptoms	BSH ingestion for 3 days did not reduce FeNO, the primary outcome, compared with placebo. Despite a marked increase in serum SFN concentrations, BS consumption did not induce cytoprotective antioxidant gene expression in PBMCs or NEC. No reduction observed in oxidative stress/inflammatory biomarkers. No improvement in lung function/respiratory symptoms detected after BS ingestion.
Egner (2014) [137]	Randomized, placebo-controlled clinical trial; 291 adults; Rural community; China; Community high pollution	BSH-derived beverage (600 μmol glucorapha- nin, 40 μmol SFN; 12 weeks) or placebo	Detoxification of air pollutants (benzene, acrolein and crotonaldehyde), GSTT1 influence	BSH beverage showed increases in urinary excretion of glutathione-derived conjugates. Statistically significant increases for benzene mercapturic acids (+61%) and acrolein mercapturic acids (+23%). No significant increase for crotonaldehyde-derived mercapturic acids. Excretion of benzene-derived methabolits was higher in GSTT1+ individuals compared with GSTT1-null, regardless of treatment.
Rosser (2025) [138]	Post hoc analysis of a double-blind, randomized, placebo-controlled; 192 asthmatic and low vit. D children; VDKA, USA; Ambient PM_2.5_ exposure	Vitamin D_3_ supplementation (4000 IU/day for 48 weeks) or placebo	Severe asthma exacerbations (6–16 years)	Children with low baseline vitamin D had higher SAE rates; Incidence of ≥1 SAE during the study was similar between treatment arms; vitamin D levels significantly inversely correlated with annual PM_2.5_ exposure; Multivariable analysis showed a significant interaction between vitamin D supplementation and PM_2.5_ exposure in relation to SAEs.
Forno (2020) [139]	Randomized, double-blind, placebo-controlled; VDKA; USA 192 children with persistent asthma and low vit. D levels (<30 ng/mL), aged 6–16 years	Vitamin D_3_ supplementation (4000 IU/day for 48 weeks) or placebo	Time to (viral induced) severe asthma exacerbation. Corticosteroid dose Serious adverse events	37.5% participants in the vitamin D_3_ group vs. 34.4% in placebo experienced ≥1 severe exacerbation. No significant differences in exacerbation or adverse effects. No significant differences in time to viral-induced exacerbation, corticosteroid dose reduction, or cumulative corticosteroid dose. Vitamin D_3_ supplementation did not significantly improve the time to severe asthma exacerbation in children with persistent asthma and low vitamin D.

Abbreviations: ASH, alfalfa sprout homogenate; BSE, Broccoli Sprout Extract; BSH, equivalent to 111 g of commercially available Broccosprouts^®^ (Brassica Protection Products LLC); DE, diesel exhausts; DEP, diesel exhaust particles; DRS, dose–response slope; FA, filtered air; FeNO: fractional exhaled nitric oxide; FEV_1_, forced expiratory volume in 1 s; IU, international unit; NRF2, Nuclear Factor Erythroid 2-Related Factor 2; NEC, nasal epithelial cells; PBMCs, peripheral blood mononuclear cells; PM_2.5_, particulate matter with aerodynamic diameter ≤ 2.5 μm; PMN, Polymorphonuclear; SABA, Short-Acting Beta-Agonist; Vit., vitamin; VDKA, Vitamin D Kids Asthma; SFN, sulforaphane; WBC, white blood cell counts.

## Data Availability

No new data was created.

## Supplementary Materials

The following supporting information can be downloaded at https://www.mdpi.com/article/10.3390/nu18040639/s1, Table S1: Multi-level mitigation strategies for pollution-related asthma, aligned with susceptibility modifiers.

## Abbreviations

The following abbreviations are used in this manuscript: AQGs, World Health Organization Global Air Quality Guidelines; AQI, Air Quality Index; BSE, broccoli sprout extract; BSH, broccoli sprout homogenate; CI, confidence interval; DEP, diesel exhaust particles; EDC(s), endocrine-disrupting chemical(s); ECRHS, European Community Respiratory Health Survey; EJ, environmental justice; EU, European Union; FeNO, fractional exhaled nitric oxide; FEF_25–75_, forced expiratory flow at 25–75% of FVC; FEV_1_, forced expiratory volume in 1 s; FO, fish oil; FVC, forced vital capacity; GINA, Global Initiative for Asthma; GPCR, G-protein-coupled receptor; GPR41/GPR43, G-protein-coupled receptor 41/43; G6PD, glucose-6-phosphate dehydrogenase; HDAC, histone deacetylase; HDM, house dust mite; HR, hazard ratio; IAQ, indoor air quality; IQR, interquartile range; IL-17, interleukin-17; IRR, incidence rate ratio; LPS, lipopolysaccharide; MUC5AC, mucin 5AC; NAC, N-acetylcysteine; NO_2_, nitrogen dioxide; NRF2, nuclear factor erythroid 2–related factor 2; O_3_, ozone; OBS, oxidative balance score; OO, olive oil; OR, odds ratio; PAH(s), polycyclic aromatic hydrocarbon(s); PBMC(s), peripheral blood mononuclear cell(s); PM, particulate matter; PM_2.5_, particulate matter with aerodynamic diameter ≤ 2.5 μm; PM_10_, particulate matter with aerodynamic diameter ≤ 10 μm; ppb, parts per billion; PUFA, polyunsaturated fatty acids; ROS, reactive oxygen species; SCFA(s), short-chain fatty acid(s); SFN, sulforaphane; SHS, secondhand smoke; SO_2_, sulfur dioxide; SVOC(s), semi-volatile organic compound(s); Th17, T helper 17; TRAP, traffic-related air pollution; VOC(s), volatile organic compound(s); WBC, white blood cell(s); WHO, World Health Organization; ω-3 PUFA, omega-3 polyunsaturated fatty acids; ω-6 PUFA, omega-6 polyunsaturated fatty acids.
