# Supplementary material for: Air Pollution, Asthma and Diet: From Mechanisms to Prevention Strategies
Source: Nutrients. 2026 Feb 15;18(4):639. doi: 10.3390/nu18040639 (PMC12942803; doi:10.3390/nu18040639)
Supplement: Supplementary file 1 [file nutrients-18-00639-s001.zip › nutrients-4123196-supplementary.pdf]

Table S1. Multi-level mitigation strategies for pollution-related asthma, aligned with susceptibility modifiers

Modifier	Mechanism	Policy/ community actions	Clinical actions	Household / individual actions	Evidence strength and key sources
**Life-course timing**	Rapid lung growth and immune maturation: stronger and more persistent pollutant effects in early windows	Protect pregnant people and children in high-exposure areas; reduce TRAP near schools/childcare; school IAQ standards (ventilation, dampness control)	Flag pregnancy and early childhood as high-priority for exposure counselling and asthma prevention; strengthen follow-up for early wheeze	Use alerts to reduce peak exposures where feasible; prioritise smoke-free environments; avoid irritant cleaning products around infants	O (strong) for early-life vulnerability; G/P for child-focused prevention [1-6]
**Environmental inequality / socioeconomic vulnerability**	"Multiple hit" model: higher exposures and lower capacity to avoid/manage → amplified morbidity	Target hotspots; equity metrics in air-quality plans; fund housing remediation and school ventilation; enforce landlord accountability	Reduce barriers to guideline-based care (access, affordability, continuity); school-based programmes; community health workers	Provide low-cost exposure-reduction options (ventilation practices, smoke-free rules, safer cleaning); avoid "responsibilities" messaging	O (strong) + equity framing; I/P essential for translation [7-15].
**Psychosocial stress and chronic adversity**	Stress and pollution synergise via oxidative and inflammatory pathways may worsen control/exacerbations	"Co-benefit" policies: housing stability, violence prevention, social protection; evaluate respiratory outcomes	Screen for psychosocial stressors; integrate support/referral; incorporate into risk stratification for peaks	Promote practical coping supports; avoid punitive avoidance advice that is not feasible	O (moderate); I (limited) for stress-reduction as effect modifier [12, 14, 16].
**Obesity as treatable trait**	Metabolic inflammation and altered oxidative balance → poorer control; may amplify pollutant responses	Structural nutrition policies (healthy school food; affordability of minimally processed foods); built environments enabling activity	Treatable-trait model: assess weight/metabolic status; tailor plans for symptom control; integrate diet quality counselling	Sustainable food-first patterning; realistic activity planning (timing away from peaks), consistent with asthma control	O (moderate–strong) for worse outcomes; O (emerging) for interaction with pollutants [17-19]
**Tobacco smoke and e-cigarette aerosols**	Potent airway irritant and inflammatory exposure; interacts with other vulnerabilities (i.e. obesity)	Smoke-free multi-unit housing protections; cessation supports; enforce smoke-free environments around children	Systematic assessment and cessation support; include in action plans; prioritise high-risk households	Smoke-free home/car rules; minimise indoor pollutant accumulation	G/O (strong) as asthma determinant; interaction evidence present [20-23].
**Indoor pollutants**	Airway irritation; epithelial barrier effects; contributes to "indoor exposome" mixtures	Ingredient disclosure/limits for high-irritant VOCs/fragrances; school/workplace guidance; safer procurement policies	Ask specifically about cleaning sprays/fragrances; counsel on reduction/substitution; integrate into trigger review	Ventilate during/after cleaning; avoid mixing products; avoid cleaning around children; use diluted concentrations when appropriate	G/O (moderate) with mixture/synergy emphasis [20].
**Dampness / mould**	Chronic indoor inflammatory exposure; co-exposure with other indoor pollutants/allergens	Enforce housing standards; remediation funding; minimum ventilation/moisture control; landlord accountability	Screen for dampness/mould; document for referrals; prioritise high-risk patients	Moisture control + prompt leak repair; ensure ventilation (esp. bathrooms/kitchens)	G/O (moderate–strong) for asthma outcomes; high policy relevance [20].
**Aeroallergens as co-triggers**	Pollutants may potentiate allergen-driven inflammation: exacerbations in sensitised individuals	Improve IAQ in schools/housing; integrate allergen seasons into public health communications where relevant	Link allergen seasons and pollution peaks in action plans for sensitised patients; optimise controller therapy	Timing of outdoor activity during peak pollen/pollution; indoor practices for allergen reduction where feasible	O (moderate); mechanistic plausibility + clinical relevance [20].
**Diet quality**	Oxidative stress buffering and immune modulation; diet may attenuate pollution-related lung function decrements and asthma	Improve access/affordability of minimally processed foods; school meals supporting fruit/veg/whole grains	Frame diet as susceptibility modifier, not substitute for emissions reduction; integrate into obesity/treatable-trait care	Emphasise fruits/vegetables/legumes/whole grains; healthier fats; reduce ultra-processed foods (pragmatic targets)	O (emerging) for effect modification; heterogeneity acknowledged [18, 24-28].
**Nutritional Supplements**	Specific pathways: lipid mediators, redox biology, microbiome–immune crosstalk; trial evidence mixed by endpoint/formulation	Not a primary policy lever; consider dietary guideline alignment (fish/healthy fats; fibre availability)	Use selectively (e.g., deficiency correction; context-specific); avoid population-wide "pill solution" framing	Prefer food sources; supplementation only when clinically indicated/justified; recognise endpoint-dependent evidence	E (mixed/endpoint-specific); O (supportive); L for some interventions [29-36]

**Risk communication / AQI alerts**	Can support behavioural adaptation during peaks; effectiveness depends on access/feasibility	Design inclusive alerts; integrate with schools/health systems; provide actionable guidance; avoid widening inequities	Incorporate AQI into written action plans for those likely to benefit; prioritise severe/poorly controlled asthma	Adjust timing/location of activities when feasible; improve indoor practices during peaks	I (variable); depends on implementation and equity [37, 38].
**Mixtures and exposome framing**	Single-pollutant thinking misses synergistic indoor–outdoor mixtures; heterogeneity driven by co-exposures	Move towards mixture-aware standards/monitoring; research and surveillance that captures indoor/outdoor co-exposures	Treatable-traits and co-exposure assessment; avoid one-size-fits-all counselling	Bundle feasible actions across exposures (smoke, dampness, irritants, diet)	Conceptual + G support; methods still evolving [16, 20].

Abbreviations: AQGs, World Health Organization Global Air Quality Guidelines; AQI, Air Quality Index; EJ, environmental justice; GINA, Global Initiative for Asthma; HDAC, histone deacetylase; IAQ, indoor air quality; OBS, oxidative balance score; PM, particulate matter; $PM_{2.5}$, particulate matter with aerodynamic diameter ≤2.5μm; PM_{10}, particulate matter with aerodynamic diameter ≤10μm; PUFA, polyunsaturated fatty acids; ROS, reactive oxygen species; SCFA, short-chain fatty acids; SHS, second-hand smoke; TRAP, traffic-related air pollution; VOC, volatile organic compounds; SVOC, semi-volatile organic compounds. Evidence strength shorthand: G, guideline; P, policy; O, observational; E, experimental/clinical trial; I, implementation/behavioural effectiveness; L, limited/heterogeneous.

1. Bettiol, A., et al., *The first 1000 days of life: traffic-related air pollution and development of wheezing and asthma in childhood. A systematic review of birth cohort studies.* Environ Health, 2021. **20**(1): p. 46.
2. Bové, H., et al., *Ambient black carbon particles reach the fetal side of human placenta.* Nat Commun, 2019. **10**(1): p. 3866.
3. Hehua, Z., et al., *The impact of prenatal exposure to air pollution on childhood wheezing and asthma: A systematic review.* Environ Res, 2017. **159**: p. 519-530.
4. Veras, M.M., et al., *Before the first breath: prenatal exposures to air pollution and lung development.* Cell Tissue Res, 2017. **367**(3): p. 445-455.
5. Yan, W., et al., *The impact of prenatal exposure to PM(2.5) on childhood asthma and wheezing: a meta-analysis of observational studies.* Environ Sci Pollut Res Int, 2020. **27**(23): p. 29280-29290.
6. Zanobetti, A., et al., *Early-Life Exposure to Air Pollution and Childhood Asthma Cumulative Incidence in the ECHO CREW Consortium.* JAMA Netw Open, 2024. **7**(2): p. e240535.
7. Byrwa-Hill, B.M., et al., *Living in environmental justice areas worsens asthma severity and control: Differential interactions with disease duration, age at onset, and pollution.* J Allergy Clin Immunol, 2023. **152**(5): p. 1321-1329.e5.
8. Cacciatore, S., et al., *Urban health inequities and healthy longevity: traditional and emerging risk factors across the cities and policy implications.* Aging Clin Exp Res, 2025. **37**(1): p. 143.
9. de Castro Mendes, F., et al., *Household Food Insecurity, Lung Function, and COPD in US Adults.* Nutrients, 2021. **13**(6).
10. Grunwell, J.R., et al., *Environmental Injustice Is Associated With Poorer Asthma Outcomes in School-Age Children With Asthma in Metropolitan Atlanta, Georgia.* J Allergy Clin Immunol Pract, 2024. **12**(5): p. 1263-1272.e1.
11. Landry, F., J. Dupras, and C. Messier, *Convergence of urban forest and socio-economic indicators of resilience: A study of environmental inequality in four major cities in eastern Canada.* Landscape and Urban Planning, 2020. **202**: p. 103856.
12. Paciência, I., J. Cavaleiro Rufo, and A. Moreira, *Environmental inequality: Air pollution and asthma in children.* Pediatr Allergy Immunol, 2022. **33**(6).
13. Redmond, C., et al., *Socioeconomic disparities in asthma health care utilization, exacerbations, and mortality: A systematic review and meta-analysis.* J Allergy Clin Immunol, 2022. **149**(5): p. 1617-1627.
14. Willis, M.D., et al., *Changes in Socioeconomic Disparities for Traffic-Related Air Pollution Exposure During Pregnancy Over a 20-Year Period in Texas.* JAMA Network Open, 2023. **6**(8): p. e2328012-e2328012.
15. Zahnow, R., et al., *Climate change inequalities: A systematic review of disparities in access to mitigation and adaptation measures.* Environmental Science & Policy, 2025. **165**: p. 104021.
16. Union, E.U.J.O.J.E., *EU Directive (EU) 2024/2881 of the European Parliament and of the Council of 23 October 2024 on Ambient Air Quality and Cleaner Air for Europe (Recast).* 2024.
17. Althoff, M.D., et al., *Obesity-related Asthma: A Pathobiology-based Overview of Existing and Emerging Treatment Approaches.* Am J Respir Crit Care Med, 2024. **210**(10): p. 1186-1200.
18. Mendes, F.C., V. Garcia-Larsen, and A. Moreira, *Obesity and Asthma: Implementing a Treatable Trait Care Model.* Clin Exp Allergy, 2024. **54**(11): p. 881-894.
19. Wong, M., E. Forno, and J.C. Celedón, *Asthma interactions between obesity and other risk factors.* Ann Allergy Asthma Immunol, 2022. **129**(3): p. 301-306.
20. Agache, I., et al., *EAACI Guidelines on Environmental Science for Allergy and Asthma-Recommendations on the Impact of Indoor Air Pollutants on the Risk of New-Onset Asthma and on Asthma-Related Outcomes.* Allergy, 2025. **80**(3): p. 651-676.
21. Castro-Rodriguez, J.A., et al., *Risk and Protective Factors for Childhood Asthma: What Is the Evidence?* J Allergy Clin Immunol Pract, 2016. **4**(6): p. 1111-1122.
22. Kitsantas, P. and F. Aguisanda, *Association of asthma with obesity among adolescents exposed to environmental tobacco smoke.* J Asthma, 2016. **53**(1): p. 25-9.
23. Wu, T.D., et al., *Overweight/obesity enhances associations between secondhand smoke exposure and asthma morbidity in children.* J Allergy Clin Immunol Pract, 2018. **6**(6): p. 2157-2159.e5.
24. de Castro Mendes, F., et al., *The inflammatory potential of diet impacts the association between air pollution and childhood asthma.* Pediatr Allergy Immunol, 2020. **31**(3): p. 290-296.
25. Frontela-Saseta, C., et al., *Ultra-processed foods consumption and asthma in the Western diet.* Dietetics, 2024. **3**(2): p. 144-158.
26. Lim, C.C., et al., *Mediterranean Diet and the Association Between Air Pollution and Cardiovascular Disease Mortality Risk.* Circulation, 2019. **139**(15): p. 1766-1775.
27. Romieu, I., et al., *Dietary intake, lung function and airway inflammation in Mexico City school children exposed to air pollutants.* Respir Res, 2009. **10**(1): p. 122.
28. Brigham, E., A. Hashimoto, and N.E. Alexis, *Air Pollution and Diet: Potential Interacting Exposures in Asthma.* Curr Allergy Asthma Rep, 2023. **23**(9): p. 541-553.
29. Li, L., et al., *Cardiorespiratory effects of indoor ozone exposure during sleep and the influencing factors: A prospective study among adults in China.* Sci Total Environ, 2024. **924**: p. 171561.
30. Chang-Chien, J., et al., *Vitamin D ameliorates particulate matter induced mitochondrial damages and calcium dyshomeostasis in BEAS-2B human bronchial epithelial cells.* Respir Res, 2024. **25**(1): p. 321.
31. Pfeffer, P.E., et al., *Effects of vitamin D on inflammatory and oxidative stress responses of human bronchial epithelial cells exposed to particulate matter.* PLoS One, 2018. **13**(8): p. e0200040.
32. Moreno-Macías, H., et al., *Ozone exposure, vitamin C intake, and genetic susceptibility of asthmatic children in Mexico City: a cohort study.* Respir Res, 2013. **14**(1): p. 14.
33. Lin, H., et al., *Consumption of fruit and vegetables might mitigate the adverse effects of ambient PM(2.5) on lung function among adults.* Environ Res, 2018. **160**: p. 77-82.
34. Duran, C.G., et al., *A proof-of-concept clinical study examining the NRF2 activator sulforaphane against neutrophilic airway inflammation.* Respir Res, 2016. **17**(1): p. 89.
35. Carlsten, C., et al., *Anti-oxidant N-acetylcysteine diminishes diesel exhaust-induced increased airway responsiveness in person with airway hyper-reactivity.* Toxicol Sci, 2014. **139**(2): p. 479-87.
36. Sudini, K., et al., *A Randomized Controlled Trial of the Effect of Broccoli Sprouts on Antioxidant Gene Expression and Airway Inflammation in Asthmatics.* J Allergy Clin Immunol Pract, 2016. **4**(5): p. 932-40.
37. Rosser, F.J., et al., *Vitamin D Supplementation, Long-Term PM(2.5) Exposure, and Severe Asthma Exacerbations in Children with Low Vitamin D: A Post Hoc Analysis of a Double-Blind, Randomized, Placebo-controlled Trial (VDKA).* Am J Respir Crit Care Med, 2025. **211**(2): p. 266-268.
38. Scholar, W.H.O.J.G., *WHO global air quality guidelines: particulate matter (PM2. 5 and PM10), ozone, nitrogen dioxide, sulfur dioxide and carbon monoxide. Geneva: World Health Organization.* 2021.
